# Environmental applications of magnetic nanohybrid materials

**DOI:** 10.1039/d5ra03470a

**Published:** 2025-06-11

**Authors:** Sidra Pervaiz, Mohsin Javed, Afzal Shah, Ansa Latif, Sidra Nasir, Iltaf Shah

**Affiliations:** a Department of Chemistry, Government College University Faisalabad 38040 Pakistan; b Department of Chemistry, Quaid-i-Azam University Islamabad 45320 Pakistan afzals_qau@yahoo.com; c Department of Chemistry, University of Agriculture Faisalabad 38000 Pakistan; d Department of Applied Chemistry, Government College University Faisalabad 38040 Pakistan; e Department of Chemistry, College of Science, United Arab Emirates University Al Ain P. O. Box 15551 United Arab Emirates altafshah@uaeu.ac.ae

## Abstract

The distinctive characteristics and versatile functionalities of magnetic nanoparticles (MNPs) position them as promising candidates for numerous environmental applications, attributed to their structural and physiochemical properties. Magnetic nanoparticles can integrate organic polymers, carbon-based materials, and metal oxides to form multifunctional composites that effectively adsorb and degrade pollutants. This review article discusses the magnetic nanomaterials and the techniques employed to functionalize and tailor these materials and their application for wastewater treatment, cleanup of oil spills, and photocatalytic degradation of pollutants of emerging concern. Focusing on their regeneration capabilities, catalytic performance, and adsorption efficiency, the article analyzes how magnetic nanohybrids engage with pollutants remediation. Additionally, it evaluates these materials' stability, implications, and recyclability to assess their practical applicability in real-world environmental scenarios. By presenting a detailed overview of the recent advancements, challenges, and prospects, this work aims to assist researchers in developing and enhancing magnetic nanohybrids as a remediation technology to advance sustainable environmental remediation strategies. The magnetic nanoparticles discussed in this document exhibit remarkable efficacy in contaminant removal due to their magnetic retrievability, facilitating easier recovery and reuse. These advantages not only enhance their practical applications but also align with principles of sustainability and resource efficiency. The innovative integration of advanced magnetic materials with green chemistry principles outlined in this review significantly enhances environmental remediation initiatives. This review adopts a holistic approach by emphasizing the benefits while addressing challenges, including instability, elevated costs, quality and functionality problems, concerns regarding secondary pollution, and issues related to reusability.

## Abstract 2

The distinctive characteristics and versatile functionalities of magnetic nanoparticles (MNPs) position them as promising candidates for numerous environmental applications, attributed to their structural and physiochemical properties.

## Graphical abstract

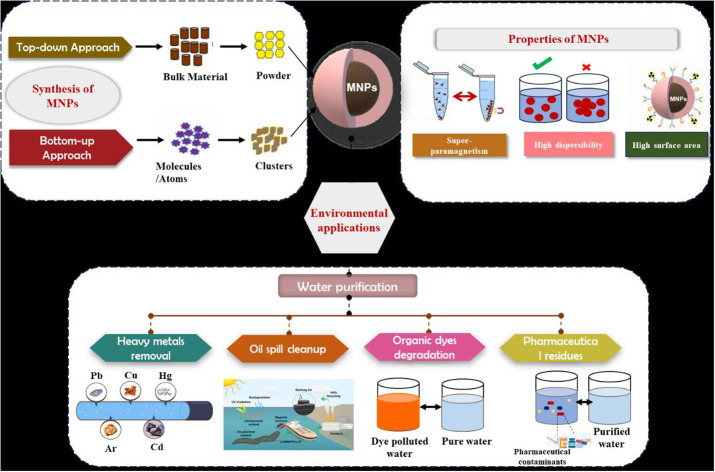

## 1. Introduction

The global population has been increasing consistently with urban areas experiencing more significant population growth, attributed to enhanced healthcare, educational opportunities, and job accessibility, with industrialization playing a key role in this urbanization process.^1^ As the population increases, the need for goods and services rises, leading to industrial growth and additional factories.^2^ The need to support larger populations and urban centers drives technological innovations in manufacturing, energy production and transportation. Many industrial and non-industrial sources contribute to water pollution, which harms human health,^3^ aquatic ecosystems, and biodiversity.^4,5^ The chemical manufacturing, mining, oil, and textile sectors discharge toxic materials into water bodies through untreated wastewater, unintentional spills, and poor waste disposal.^6^ These substances include organic pollutants^7^ like solvents, dyes, hydrocarbons, and heavy metals^8^ such as lead, mercury, and arsenic. Moreover, agricultural runoff comprising fertilizers, pesticides, and animal manure causes eutrophication, toxic algal blooms, and pathogens^9^ contamination, in addition to deforestation and soil erosion.^10^ Furthermore, mining results in metal leaching and acid mine drainage,^11^ and hydraulic fracturing in oil and gas operations puts groundwater at risk of contamination.^12^ Marine environments face threats from plastic waste, oil spills, and the atmospheric deposition of pollutants such as nitrogen oxides.^13^ To protect water quality and ecosystem health, integrated legislation, sustainable practices, and efficient wastewater treatment technologies must be implemented.^14^ Managing the impact of urbanization and industrialization is crucial for environmental sustainability.

In the realm of water and wastewater treatment, nanotechnology stands out as a highly effective and promising avenue for advancement.^15^ The utilization of magnetic nanoparticles (MNPs) has shown significant benefits in wastewater remediation, owing to their exceptional optical, electrical, and magnetic properties, as well as their high adsorption capacities, mobility, reactivity, and catalytic abilities. Furthermore, the features and applications of MNPs can be tailored through the application of magnetic fields, enhancing their efficacy in treatment processes. MNPs find extensive potential applications in catalysis, biomedicine, tissue targeting, colloidal photonic crystals, MRI, MPI, microfluidics, data storage, environmental remediation, and more.^16,17^ MNPs of many kinds, including iron oxides (Fe_3_O_4_), spinel ferrite (CoFe_2_O_4_), bio-metallic, carbon-based, and others are employed for wastewater treatment. With the potential for nearly 100% *in situ* treatment, MNPs in waste treatment improve efficiency, encourage reuse, and save money, time, and effort.^18^

Physical and chemical methods are essential for wastewater treatment. Physical methods involve mechanical techniques such as screening, filtration, and sedimentation to eliminate suspended solids and debris. Additionally, flotation and centrifugation are employed to separate materials based on density. Chemical methods utilize various substances to precipitate, neutralize, or oxidize contaminants. Coagulation and flocculation processes introduce chemicals that promote the aggregation of smaller particles into larger ones, improving their removal through sedimentation or filtration.^19^ Disinfection techniques such as chlorination and ozone treatment eliminate harmful pathogens. Chemical oxidation can break down complex organic pollutants into simpler compounds, often necessitating pH adjustments to optimize these processes. While these methods are effective, they can be costly, require careful handling of hazardous materials, and generate chemical sludge that must be disposed appropriately. Nevertheless, the implementation of both physical and chemical treatments is crucial for the effective removal of diverse pollutants, particularly in industrial wastewater.^20^ Wastewater treatment utilizing physical and chemical techniques typically involves considerable expenses and substantial energy consumption, especially when dealing with large volumes. Applying these methods often requires the use of chemicals, which can pose environmental and safety risks, in addition to generating chemical sludge that requires proper disposal. Moreover, these methods may not effectively remove certain dissolved pollutants or biodegradable organic materials, leading to the need for further treatment processes.^21^

Membrane technologies are advanced methods employed in wastewater treatment, leveraging selective barriers to efficiently separate contaminants from water. Commonly utilized membrane processes include nanofiltration, microfiltration, ultrafiltration, and reverse osmosis, each characterized by specific pore sizes and varying effectiveness in pollutant removal. Microfiltration and ultrafiltration are adept at removing suspended solids, bacteria, and larger particles, while nanofiltration and reverse osmosis target smaller dissolved contaminants, such as salts, heavy metals, and organic molecules.^22^ Nonetheless, membrane technologies can incur significant expenses owing to the energy requirements for sustaining pressure across the membrane and the regular necessity for cleaning or replacing membranes due to fouling. Membrane fouling diminishes efficiency and requires regular cleaning or replacement, leading to increased downtime and operational costs. Furthermore, this technology might only be financially viable for extensive applications with considerable investment.^23^

Among the advanced oxidation processes (AOPs), photocatalysis stands out due to its effectiveness and versatility in wastewater treatment, surpassing methods like ozone oxidation, Fenton's reagent, and UV/H_2_O_2_. A notable advantage of photocatalysis is its ability to operate under ambient conditions, which negates the necessity for high temperatures or pressures, thus streamlining operations and reducing energy costs.^24^ Unlike chemical methods like Fenton's reagent that rely on iron salts and hydrogen peroxide, photocatalysis does not require potentially harmful chemical additives, resulting in fewer secondary pollutants and a reduced need for subsequent neutralization processes.^25^ The issue of electron–hole recombination in photocatalysis, which can restrict the efficiency of the process, is currently under investigation. Innovations like heterojunction photocatalysts and plasmonic materials contribute to the reduction of recombination and enhance overall performance.^26^

Magnetic nanoparticles constitute a distinctive category of nanomaterials utilized in photocatalytic wastewater treatment. They provide specific advantages and functionalities that differentiate them from traditional photocatalysts such as TiO_2_, ZnO, CdS, and graphene-based materials.^27^ Magnetic nanoparticles, primarily consisting of iron oxides such as Fe_3_O_4_ or γ-Fe_2_O_3_, demonstrate significant magnetic properties that facilitate their separation from treated water by applying an external magnetic field. This feature enhances the recovery and reuse of photocatalysts, tackling a considerable challenge of conventional photocatalysts, which often face time-consuming, costly, or inefficient separation from treated effluent. Materials such as TiO_2_ and ZnO demonstrate high effectiveness; however, their recovery usually necessitates filtration or sedimentation, potentially elevating operational costs. Magnetic nanoparticles can be rapidly and effectively extracted using magnetic fields, enhancing the overall process's economy and scalability, particularly in large-scale wastewater treatment applications.^28^ MNPs frequently act as a support or core material for other semiconductors, improving their photocatalytic activity. A photocatalyst such as TiO_2_ or ZnO can be applied to the surface of MNPs, resulting in a composite that utilizes the photocatalytic characteristics of the semiconductor while also providing the benefit of magnetic separation. This hybrid method integrates the advantageous characteristics of both materials: the elevated photocatalytic efficiency of semiconductors and the straightforward recovery of magnetic particles.^29^ MNPs contribute to the reduction of electron–hole recombination in these systems, a process where electrons and holes recombine, reducing the efficiency of the photocatalyst, as the iron oxide core facilitates electron transfer, enhancing overall photocatalytic performance. This contrasts with conventional semiconductor materials such as TiO_2_, ZnO, or CdS, where recombination remains a significant challenge restricting reactive species' production. TiO_2_ is susceptible to electron–hole recombination; however, incorporating magnetic nanoparticles as a support material can enhance charge separation and increase the efficiency of the photocatalyst.^30^ MNPs display distinctive surface chemistry that can be utilized for additional functionalization. Modification or doping with metals, oxides, or organic molecules can enhance catalytic properties, improve light absorption, or introduce specific functionalities for pollutant binding. Combining Fe_3_O_4_ nanoparticles with silver or gold nanoparticles enhances light absorption. It broadens their activity into the visible spectrum, thereby overcoming a significant limitation of conventional photocatalysts such as TiO_2_, which predominantly function under UV light.^31^

Magnetic nanoparticles enhance process efficiency by facilitating improved charge separation and easier recovery post-treatment rather than directly influencing photocatalytic reactions. This renders them more complementary, in contrast to traditional photocatalysts designed to degrade pollutants independently.^32^ The interaction between MNPs and traditional photocatalysts presents a promising approach for enhancing wastewater treatment solutions' efficiency, sustainability, and cost-effectiveness.^18^ This review explores MNPs, focusing on their structural characteristics and photocatalytic capabilities, while highlighting their unique benefits in addressing different contaminants in wastewater. It examines surface modification and composite strategies employed to enhance the photocatalytic efficiency of magnetic nanoparticles, focusing on prevalent issues such as electron–hole recombination and material stability. This document also evaluates the versatility of these nanoparticles in addressing various pollutants, organic dyes, heavy metals, synthetic drugs, and oil spills across diverse environmental conditions. The review offers insights into recent advancements and applications, aiming to facilitate future innovations using magnetic nanoparticles for scalable and environmentally friendly wastewater treatment technologies. This is the first report that presents the regeneration capabilities, the photocatalytic performance, and adsorption efficiency of magnetic nanoparticles as a remediation technology to advance the renewable and sustainable water treatment strategies. It offers insights into recent advancements and applications, aiming to facilitate future innovations using magnetic nanoparticles for scalable and environmentally friendly wastewater treatment technologies.

## 2. Synthesis techniques for MNPs

**Fig. 1:**
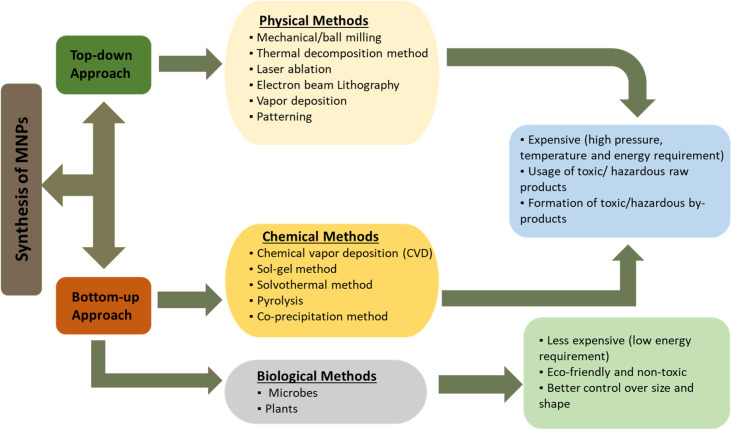
Classification of synthesis methods for MNPs and their key features.

MNPs are synthesized by various methods. In general, the synthesis of MNPs can be broadly classified into three categories (a) physical, (b) chemical, and (c) biological or microbial methods.^33^ Physical methods are based on the top-down strategy, *i.e.*, synthesis starts from bulk material and depletes to generate NPs. Chemical and biological methods follow a bottom-up or constructive approach, where the atoms/molecules assemble into different sizes of NPs.^34^Fig. 1 shows different synthesis methods of MNPs.

### 2.1 Top-down synthesis

This approach employs a destructive method to reduce bulk materials into tiny, nanoscale particles through various physical and chemical procedures. It can be demonstrated by destructive techniques such as physical vapor deposition, grinding, milling, or several others.^35^

#### 2.1.1 Mechanical method/ball milling method

This method of producing nanoparticles from bulk is cost-effective. It entails breaking down bulk materials directly into nanostructures. During mechanical milling, the bulk material receives the rollers which reduces grain size. Alloys of various metals are produced using this method.^36^ In a study by Bououdina *et al.*, nanocrystalline magnesium ferrite was synthesized through ball milling of α-Fe_2_O_3_ and MgO in powdered form and strengthened at 700–900 °C. The synthesized magnetic nanopowders were suitable for removing heavy metals from wastewater.^37^ In another study, carbonaceous magnetic adsorbents were synthesized through ball milling, a combination of activated carbon and Fe_3_O_4_ nanoparticles. The authors found that the ball-milled magnetic adsorbents are environmentally friendly to synthesize, have high efficiency, low cost, and offer convenience in operation.^38^

#### 2.1.2 Thermal decomposition method

Thermal decomposition is an endothermic process where heat causes chemical decomposition and breaks the chemical link in a compound. The nanoparticles result from the decomposition of metal at a particular decomposition temperature at which an element undergoes chemical breakdown.^39^ Hyeon and co-workers have also used a similar thermal decomposition approach to prepare monodisperse iron oxide nanoparticles. They used nontoxic and inexpensive iron(iii) chloride and sodium oleate to generate an iron oleate complex *in situ*, which was then decomposed at temperatures between 240 and 320 °C in different solvents, such as 1-hexadecene, octyl ether, 1-octadecene, 1-eicosene, or trioctylamine.^40^ The thermal decomposition of zero-valent metal precursor Fe(CO)_5_ leads to the formation of metal NPs, but if oxidation occurs, it may form high-quality iron oxide MNPs. On the other hand, if the decomposition of precursors occurs with cationic metal centers, it can directly form metal oxide NPs.^41^ This method was successfully generalized for synthesizing other magnetic nanocrystals, such as MnO, Co_3_O_4_, and NiO.^42^

#### 2.1.3 Laser ablation

This method involves laser irradiation to reduce particle size to nanoscale. A solid surface is exposed to a laser beam, creating a low-flux plasma plume that is eventually sublimated or evaporated to create NPs. The materials undergo plasma conversion at higher flux. Due to its high yield, improved long-term stability, and improved particle size and shape control, this technique is a better substitute for traditional chemical procedures.^43^ Laser ablation has also synthesized magnetic nanoparticles from transition metals and metal alloys.^44^ Using the laser ablation method, Sakiyama *et al.* fabricated Ni particles of a selected size coated with a NiO shell. The oxidation of Ni nanoparticles successfully generated a Ni/NiO core–shell structure.^45^ Shinde *et al.* also synthesized iron oxides and strontium ferrite with laser ablation and evaluated the magnetic characteristics.^46^

#### 2.1.4 Electron beam lithography

In the electron beam lithography process, iron particles are transformed into iron oxides (Fe_3_O_4_) using an electron beam, or e-beam. This process produces nanoscale iron oxide nanoparticles (NPs) by using specified e-beam emission across an iron particle-film surface. This approach is acknowledged as being inexpensive, versatile, and capable of producing stable NPs.^18,47^

#### 2.1.5 Vapour deposition and patterning

This technique can produce magnetic particles by filling holes in a template and creating a continuous film. Vapour deposition methods (laser ablation, sputtering, and evaporation) and electrodeposition methods are used to deposit the material that makes up the particles. This process has demonstrated the ability to generate high-quality NPs, lower alloying temperatures, and potentially be used on a wide scale.^18,48^

### 2.2 Bottom-up synthesis

Bottom-up synthesis, the constructive method, uses chemical and biological processes to construct MNPs from relatively more straightforward substances such as atoms, clusters, and molecules. This process initially creates the nanostructured building blocks of nanoparticles, which are subsequently put together to produce the finished product (MNPs). The final product offers a more uniform shape (physical attributes), size, and chemical composition. Chemical vapor deposition, sol–gel, and pyrolysis are examples of bottom-up synthesis.^49^

#### 2.2.1 Chemical vapor deposition (CVD)

In this method, the thin film of the gaseous reactant and a mixture of additional gas molecules are deposited onto a substrate that promotes substrate superheating. The ions are reduced when the combined gases and substrate come into contact. The outcome of this reaction is typically a film from which the MNPs must be scrapped. The production of intricate, robust, homogeneous, and highly pure NPs is a prominent feature of the CVD method. This technique is now highly significant in the semiconductor and electronics industries. However, the production of extremely hazardous gases as byproducts and the demand for specialized equipment are the main drawbacks of this method.^50^ Niu *et al.* synthesized well-defined core/shell type single crystalline Fe_7_S_8_/Fe_3_O_4_ coated α-Fe hybrids (Fe_7_S_8_/Fe_3_O_4_@Fe) with vacuum CVD technique. The CVD process triggers the conversion of naturally formed Fe_3_O_4_ layer on the surface of commercial Fe nanoparticles from amorphous into a single crystalline phase.^51^ Another study used a room-temperature method to synthesize Ni, Ni/Zn, and Zn ferrite nanoparticles. Various shapes for the produced carbon structures have been observed using ferrite nanoparticles as a growth catalyst of the CVD process to prepare crystalline MNPs.^52^

#### 2.2.2 Sol–gel method

The sol–gel method is one of the most favored straightforward techniques for synthesizing magnetic nanoparticles. This method combines two words: sol (a colloid made up of solid suspended particles in continuous liquid) and gel (a solid macromolecule dissolved in solvent). An appropriate chemical solution, such as metal oxides and chlorides, is a precursor. The precursor is distributed throughout the host liquid *via* various techniques, including shaking, stirring, and sonication. The resulting solution comprises a liquid and solid phase, separated using multiple methods like centrifugation, sedimentation, and filtration to extract the nanoparticles. It is a procedure that turns sol into gel using hydrolysis and condensation.^53^ This technique works very well for creating silica-coated and iron oxide MNPs. It makes it possible to produce MNPs in large quantities with controlled sizes and distinct shapes.^54^ Mohammad *et al.* reported a novel visible-light-driven photocatalyst of TiO_2_/Au/SWCNT nanohybrid prepared by a sol–gel approach and successfully assessed for the photocatalytic degradation of the organic methylene blue. TiO_2_/Au/SWCNT nanohybrid was obtained by doping TiO_2_ lattice with Au and supporting with modified single-walled carbon nanotubes (SWCNTs).^55^ In another study, barium ferrite spinel (BaFe_2_O_4_) was synthesized through the sol–gel method, and its nanohybrids with copolymers of carbazole and pyrrole were successfully synthesized *via* mechano-chemical mixing. The spectral, thermal, morphological, and magnetic properties of the synthesized nano-magnetic materials were analyzed *via* thermal gravimetric analysis (TGA), transmission electron microscopy (TEM), and vibrating sample magnetometer (VSM) techniques.^56^

#### 2.2.3 Solvothermal method

MNPs are created using the solvothermal process, also known as hydrothermal, in aqueous fluids at temperature above 200 °C and extremely high pressures (>13 790 kPa) using autoclaves. It primarily entails the rapid nucleation and growth of newly created MNPs, resulting in pure particles with controlled morphologies. During this process, MNPs are prepared by hydrolysis and oxidation reactions. The geometry of NPs can be better controlled using this technique.^57^

The synthesis of Fe_3_O_4_ nanoparticles (NPs) with a size of 15 nm and a spherical shape is one of the impressive achievements obtained using this technique, which has been effectively applied in tumor MRI applications.^54^ Modern nanomaterials that are challenging to synthesize through traditional methods are typically prepared thermodynamically in stable and metastable states using the solvothermal approach. Moreover, producing superior crystallized monodispersed nanocrystals is one of the features of solvothermal synthesis.^58^

#### 2.2.4 Pyrolysis

Thermal decomposition is a crucial additional method for the synthesis of magnetic nanoparticles. This process of endothermic decomposition utilizes heat to break the chemical bonds within the compound, causing the precursor to decompose. After that, force it into a chemical reaction that synthesizes MNPs and other impurities in ash. Then, NPs are separated by additional processing of the obtained ash. The precursor could be in a liquid or vapor state. A laser or plasma can also be employed in place of a flame to generate a high temperature that facilitates evaporation. It is an inexpensive and efficient continuous process with a high yield.^59^ Nugroho *et al.* aimed to implement the one-pot pyrolysis for hybrid nanomaterial synthesis consisting of graphene quantum dots (GQDs) from solid citric acid and carbon dots (CDs), which were hydrothermally derived from the herbal plant extract of “*Houttuynia cordata*,” denoted as CDs mixed GQDs. The nanohybrid was structurally characterized by XPS, FT-IR, fluorescence, and UV-visible absorption spectroscopy.^60^ In another study, Fe/Fe_3_C magnetic nanohybrids were synthesized with solid-phase pyrolysis of iron(iii)-phthalocyanine. The pyrolysis temperature and synthesis duration were altered to produce a series of nanohybrids with distinct magnetic and structural properties. Nine samples were obtained with synthesis temperatures ranging from 700 °C to 1100 °C and pyrolysis duration of 5 or 15 minutes.^61^

#### 2.2.5 Coprecipitation

This technique is most accurate and efficient approach to create superparamagnetic iron oxide nanoparticles (SPIONs), which have a mean diameter of less than 50 nm. In an aqueous monophasic liquid medium, it involves chemical interactions where the development and nucleation of coherent iron hydroxide nuclei must be controlled. A significant parameter in the magnetization of MNPs is the annealing temperature, with the most promising results occurring at temperature between 900 and 1000 °C.^62^ The typical MNPs synthesized through this method are Fe_3_O_4_ or γ-Fe_2_O_3_ NPs. Manganese ferrite (MnFe_2_O_4_) NPs were produced using sodium hydroxide (NaOH) as a precipitant and ferric chloride (FeCl_3_), manganese(ii) chloride (MnCl_2_) as suppliers of metal ions.^54^

### 2.3 Biological methods

Biological synthesis is a well-established method for synthesizing MNPs using living organisms, including plants and microorganisms such as fungi, viruses, bacteria, and actinomycetes.^63^ The benefits of this method are its efficiency, eco-friendliness, and clean process. The disadvantage is its poor dispersion of the NPs.^64^ The synthesis of NPs using plant tissue, extracts, exudates, and other plant parts has become an area of great interest for researchers.^65^ For instance, particles with an average size of 60 nm ferromagnetic magnetite were reported to be biologically synthesized.^66^ A biologically synthesized Fe_3_O_4_ magnetic material was used as a catalyst in the Suzuki–Miyaura reaction and photocatalysis.^67^

### 2.4 Functionalization of MNPs

The functionalization of magnetic nanoparticles has emerged as a highly effective strategy for environmental remediation, especially in the area of photocatalytic degradation. By modifying the surfaces of these nanoparticles with photocatalytic materials, their ability to decompose organic pollutants in both water and air is significantly improved, leading to more efficient environmental cleanup processes.^68^ The use of a magnetic core facilitates the efficient recovery and reuse of nanoparticles post-treatment, thereby reducing secondary pollution. When subjected to light, these functionalized nanoparticles produce reactive oxygen species capable of breaking down harmful contaminants, including dyes, pharmaceuticals, and pesticides. This mechanism not only purifies the environment but also presents a sustainable and cost-effective approach to managing industrial and agricultural waste. Additionally, the customization of the nanoparticles' surface chemistry allows for targeted interactions with specific pollutants, enhancing the overall efficiency of the degradation process. Consequently, the functionalization of magnetic nanoparticles represents a significant advancement in utilizing photocatalytic degradation for effective environmental remediation.^69^

The key element in the development of MNP is iron oxide materials, particularly magnetite and maghemite. Various synthesis methods, such as co-precipitation, thermal decomposition, and hydrothermal synthesis, are employed to produce these nanoparticles. Each technique allows for precise control over the size, shape, and magnetic properties of the particles, which in turn affects their performance characteristics. Among these methods, the chemical precipitation of iron salts is the most widely used for creating iron oxide nanoparticles. This process involves the precipitation of iron salts under basic conditions, resulting in the formation of Fe^2+^ and Fe^3+^ salts that aggregate into nanoparticles, typically at ambient or slightly elevated temperatures.^70^ This approach is favored for its simplicity, cost-effectiveness, and scalability, making it suitable for large-scale industrial production. In contrast, thermal decomposition involves the breakdown of organometallic precursors in high-boiling organic solvents, often in the presence of surfactants, allowing for precise control over the nanoparticles' structure and size.^71^ High-performance materials that are advantageous for various applications require exceptional magnetic properties, making the intricate and expensive single-step decomposition of organometallic precursors in high-boiling organic solvents with surfactants a critical process. Hydrothermal synthesis, which involves chemical reactions at elevated temperatures and pressures within a sealed vessel containing an aqueous solution, offers remarkable control over the formation of crystals and their respective particle sizes. This production technique allows researchers to fabricate nanoparticles with outstanding magnetic saturation and stability, suitable for complex applications.^16^

MNPs can be effectively utilized in biomedical applications by incorporating organic polymers, which enhance their biocompatibility and stability while improving dispersibility. This integration typically involves coating the nanoparticles with biocompatible polymers such as polyethylene glycol (PEG), polydopamine, or chitosan. The hydrophilic nature of PEG significantly contributes to the nanoparticles' compatibility with biological environments, resulting in prolonged circulation times in biological fluids. For instance, Xu *et al.*, successfully immobilized β-Glucosidase (β-Glu) onto MNPs, creating MNP–β-Glu, which was further modified with PEG to produce MNP–β-Glu–PEG. This modification not only enhances the stability of the nanoparticles in blood serum but also boosts the enzyme's activity.^72^ PEGylated nanoparticles are designed to avoid detection by the reticuloendothelial system, making them ideal for applications in drug delivery and imaging. These PEG-coated nanoparticles serve as effective drug carriers, enabling the targeted delivery of therapeutic agents to specific tissues or tumors while employing controlled release systems to minimize side effects during treatment. The robust and stable coating of magnetic nanoparticles is derived from polydopamine, a versatile biopolymer inspired by mussel adhesive proteins. This material contains reactive groups that allow healthcare professionals to attach targeting ligands or therapeutic agents. Consequently, polydopamine-coated nanoparticles can fulfill multiple roles, including targeted drug delivery, photothermal therapy, and functioning as biosensors.^73^ Targeted cancer cell therapy is significantly improved through the precise delivery of cancer cells using polydopamine-coated Fe_3_O_4_ nanoparticles in conjunction with antibodies or peptides. These magnetic nanoparticles are enhanced by a biocompatible and biodegradable chitosan coating, a natural polysaccharide that facilitates chemical interactions with drugs, proteins, and nucleic acids. This characteristic makes chitosan an ideal candidate for applications in genetic delivery and tissue engineering. By utilizing chitosan-based coatings on nanoparticles, researchers have established a promising approach for delivering genetic material, which holds potential for advancements in gene therapy and regenerative medicine.^74^

MNPs demonstrate improved characteristics when combined with carbon-based materials like graphene oxide (GO) and carbon nanotubes (CNTs). This synergy leads to a notable increase in surface area, enhanced electrical conductivity, and greater mechanical strength. Consequently, these hybrid composites prove to be particularly effective for environmental applications, including the removal of pollutants and the treatment of wastewater.^75^ The oxygen functional groups present on GO derivatives serve as effective binding sites for magnetic nanoparticles, which, along with their adsorption capabilities and stability, define the performance of GO-coated nanoparticles. These advanced materials play a crucial role in extracting heavy metals, dyes, and organic pollutants from water sources.^76^ Research has demonstrated that Fe_3_O_4_/GO composites are effective in removing toxic heavy metals such as Pb^2+^ and Cd^2+^ from contaminated water, leveraging their magnetic recovery properties. Additionally, cylindrical CNTs are distinguished by their remarkable electrical, thermal, and mechanical characteristics, making them notable nanostructures in various applications.^77^ The integration of magnetic nanoparticles with CNTs enhances the performance of the composite system. These hybrid materials are utilized in catalytic pollutant degradation and as electrode components in energy storage applications. The Fe_3_O_4_/CNTs composites serve as efficient catalysts for degrading wastewater dyes, attributed to the high surface area and catalytic efficiency of CNTs.^78^

By functionalizing MNPs with TiO_2_ or ZnO, their photocatalytic properties are significantly improved, making them suitable for environmental remediation. These composites utilize a magnetic core that facilitates easy separation and reuse while effectively degrading organic pollutants under light irradiation. TiO_2_ is recognized as a reliable photocatalyst due to its stability when exposed to UV light, and TiO_2_/MNP composites exhibit strong degradation capabilities for various organic pollutants, including water-soluble dyes, pesticides, and pharmaceutical residues.^79^ Specifically, Fe_3_O_4_/TiO_2_ composites have proven to be effective in the photodegradation of methylene blue dye under UV light, while also allowing for convenient magnetic recovery for recycling purposes.^80^ The photocatalytic performance of ZnO is significantly enhanced under UV light, and when ZnO is applied as a coating on MNPs, their photocatalytic efficiency improves, making them suitable for environmental applications. The use of Fe_3_O_4_/ZnO composites facilitates water purification through sustainable methods, promoting the degradation of organic pollutants like phenol and bisphenol A. Furthermore, the functionalization of MNPs represents a significant advancement in nanotechnology, providing effective solutions to various application challenges. By integrating MNPs with organic polymers, carbon-based materials, and metal oxides, researchers are addressing specific needs in both medical and environmental fields.^81^

### 2.5 Stabilization of nanoparticles

Nanoparticles possess a high surface area-to-volume ratio, which contributes to their inherent instability and susceptibility to aggregation, oxidation, or dissolution over time. This instability is further influenced by factors such as surface charge, ionic strength, pH, temperature, and surface chemistry. Consequently, stabilizing nanoparticles is crucial to maintain their unique properties, including enhanced reactivity, capacity, and optical characteristics. If these properties are compromised, the nanoparticles may become ineffective or behave unpredictably in various applications. Effective stabilization ensures that nanoparticles can be reliably stored, transported, and utilized in fields such as medicine, catalysis, and environmental science.^82^ When nanoparticles aggregate, they form tightly bound collections that are difficult to separate into individual particles, causing them to behave more like bulk materials and resulting in a loss of their unique qualities, such as high reactivity or catalytic efficiency. By implementing stabilization techniques, aggregation is prevented, allowing the nanoparticles to retain their distinct and active characteristics.^83^

Chemically synthesized nanoparticles are typically stabilized by surface-capping ligands that control their final dimensions. In the absence of these stabilizing agents, van der Waals forces would cause the colloidal particles to aggregate and precipitate. Stabilizers or capping agents counteract this tendency by creating a repulsive force between the particles. For instance, charged capping agents like sodium citrate facilitate electrostatic stabilization, where negatively charged citrate-capped nanoparticles attract positively charged counter-cations from the surrounding medium, resulting in the formation of an electrical double layer that induces coulombic repulsion. Alternatively, steric stabilization is achieved through the use of long-chain thiols or larger molecules such as polymers. Various stabilization methods exist, including the chemical binding of stabilizing molecules to the nanoparticle surface, the introduction of particle charges, or the creation of physical barriers to prevent aggregation.^84^

#### 2.5.1 Electrostatic stabilization

Electrostatic stabilization originates from the repulsive forces experienced by nanoparticles when they are surrounded by a double layer of electric charges, which effectively prevents their agglomeration. This stabilization process involves charging the surfaces of nanoparticles through the introduction of ions, acids, or bases, or by ionizing surface functional groups at specific pH levels, thereby minimizing the risk of aggregation.^85^ The pH of the surrounding medium plays a crucial role in determining the ionization state of these functional groups, thereby influencing the surface charge of the nanoparticles. Optimal stability is observed at a specific pH level, where increased deprotonation of surface groups leads to a higher negative charge on the nanoparticles. This enhanced negative charge promotes greater repulsion between particles, thereby improving their stability. The zeta potential is a key measure of the effective surface charge and potential stability of nanoparticles.^86^ A low zeta potential indicates inadequate stability and a likelihood of particle aggregation, while a high zeta potential, typically exceeding ±30 mV, suggests strong repulsive forces among particles. The pH level can influence the dispersion characteristics by altering the zeta potential or surface charge. In aqueous and colloidal suspensions, electrostatic stabilization plays a crucial role in maintaining stability.

#### 2.5.2 Steric stabilization

Steric stabilization refers to the mechanism by which large molecules, including polymers and surfactants, adhere to the surfaces of nanoparticles to prevent their aggregation.^85^ As the number of these adsorbed molecules rises, the osmotic repulsive forces also increase, thereby improving the stability of the nanostructured particles. The repulsive forces that facilitate steric stabilization can be understood by examining the extended interaction potential. Factors such as temperature, average chain length, polymer concentration, and solubility influence steric stability, which remains unaffected by particle size due to its nature as a short-range interaction.^87^

#### 2.5.3 Electrosteric stabilization

Electro-steric stabilization refers to the synergistic effect of electrostatic and steric stability,^88^ which is achieved by adsorbing ionic polymers onto the surfaces of charged particles. This process generates both electrostatic and steric barriers that effectively inhibit the agglomeration of dispersed nanoparticles. The electrostatic double layer formed through this technique significantly improves the dispersion of nanofluids compared to methods relying solely on steric repulsion. Typically, this approach employs polyelectrolyte polymers, which consist of repeating units linked by at least one ionized functional group, such as carboxylic or sulfonic acid groups. Commonly used anionic polyelectrolytes include polyacrylic acid, poly(styrene sulfonic acid), and poly(1-vinylpyrrolidone-*co*-acrylic acid), while cationic options like polyethyleneimine and poly(vinyl sulfonic acid) are also utilized for the electro-steric stabilization of nanoparticles in suspension.^89^ The ionized functional groups attach to the polymers and are distributed within the base fluid, forming an electrical double layer around incoming particles. For effective stabilization, it is essential that the ionic polyelectrolytes and nanoparticles possess opposite charges. Additionally, polyampholytes, which feature both anionic and cationic functional groups, can be utilized for electro-steric stabilization. Ionic liquids contribute to the stabilization of nanoparticles by offering both electrostatic repulsion and steric hindrance, primarily due to their high charge density and strong chemisorption on the NP surface. Table 1 outlines the pros and cons of various synthesis techniques for magnetic nanohybrid materials.

**Table 1:** Comparison of the synthesis methods

Synthesis method	Cost	Size control	Yield	Scalability	Advantages	Disadvantages	Ref.
Mechanical ball milling	Low	Good	High	High	Wide particle size distribution, ease of operation, high efficiency	Irregular shape, high energy consumption and less production efficiency	90
Thermal decomposition	High	Excellent	Low	High	Very narrow size distribution, highly scalable and good shape control	Complicated synthesis, inert atmosphere and high temperature requirement	54
Laser ablation	High	Good	Low	Low	Narrow size distribution, monodispersity, simple & efficient, high purity & crystallinity	High input energy & uneconomical	91
Electron beam lithography	High	Fine	Low	Low	Well controlled inter-particle spacing	Expensive & extremely costly machinery needed	18
Chemical vapour deposition	High	Good	Limited	High	Production of intricate, robust, homogeneous, and highly pure NPs	Production of toxic gases as byproduct, demand for specialized equipment	18 and 92
Sol gel method	Low	Good	Variable	High	Good control of particle size and microstructure, monodispersity, desirable shape and length of the products, good crystallinity and high purity	Contamination of product with matrix component, weak bonding, low wear resistance, high permeability	93 and 94
Solvothermal	High	Excellent	Marginal	High	Very narrow size distribution & high crystallinity, precise size & shape control	High reaction temperature & pressure	95
Spray pyrolysis	Low	Good	Moderate	High	Tunable magnetic properties, homogeneous coatings	High energy consumption, risk of aggregation, low production efficiency	96 and 97
Coprecipitation	Low	Poor	High	High	Simple, narrow size distribution and high production rate	Poor morphology and non-stoichiometric magnetite	98 and 99

## 3. Environmental applications of MNPs

### 3.1 Water purification using MNPs as adsorbents

Freshwater consumption is likely to increase due to the growing population. Over the past century, the demand for water has increased almost sevenfold, while the global population has risen fourfold.^100–102^ Water availability per capita is expected to decrease by 2050, increasing the likelihood of water stress.^103^ Despite 2.6 billion people gaining access to quality drinking water since 1990, projections indicate that access to water-stressed areas will be available to 3.9 billion people by 2030.^104^ Contaminants in water resources, resulting from both natural and human activities, are threatening various sectors, and the quality of these resources is declining significantly. Continued poor results in managing water quality, variations in rainfall, over-exploitation of groundwater sources, and water pollution are equally a problem, as are imbalances in the allocation of resources and the occurrence of droughts. Sewages from industries, urban/peri-urban settlements, poor disposal of wastes, traffic irrigation water, and animal wastes, especially in rural areas, play an excessive role in water pollution.^105^

#### 3.1.1 Adsorption of dyes

Carboxymethyl cellulose (CMC), a cellulose derivative, has several attributes: biodegradable, biocompatibility, hydrophilic, nontoxicity, and the least expensive. Nasiri *et al.* employed CMC to develop a nanomagnetic adsorbent, CoFe_2_O_4_@CMC/HZSM-5, to remove metronidazole (MNZ) from water. The structures of the adsorbents were characterized by using several methods. The maximum MNZ removal was obtained at optimum conditions of 6 pH, 20 °C temperature, 50 mg L^−1^ concentration of MNZ, and a reaction time of 60 minutes. The average removal efficiency was 94% for the synthetic wastewater samples and 85% for the actual wastewater samples. The adsorbent lost only 14% of its capacity in six consecutive cycles.^106^

The iron oxide-flax seed-based hybrid nanocomposites were synthesized by Choudhry *et al.* and were utilized and examined for the adsorption of malachite green. Experimental outcomes proved that the composite achieved high removal efficiency, as 90% of malachite green was obtained from a 10.0 mg L^−1^ solution, with 1.0 g L^−1^ of the composite, within 15 minutes at 30 °C and pH 7. The most time-consuming process in the adsorptive removal of malachite green on the catalyst was intraparticle diffusion, which might occur due to weak electrostatic and hydrogen bonding. In conclusion, this work shows that this magnetic flax seed-based composite is effective for water treatment using adsorption technology.^107^ Saber *et al.* synthesized two categories of nanohybrids. First, cobalt iron oxide nanoparticles were combined with Al/Zn nanolayers. The second one used long-chain fatty acids, including stearic acid, to increase the distance of Al/Zn nanolayers and create incorporation pathways for the NPs of cobalt iron oxide. Additionally, the zinc oxide nanohybrids demonstrated a lowering in band gap energy value from 3.20 to 2.75 eV, thus increasing the performance of the cells in the sunlight. This high efficiency of the nanohybrid was illustrated by the total decolorization of Acid Green 1 dye within 10 minutes of exposure to sunlight, unlike the pure and doped ZnO, which took 360–840 minutes. The pseudo-first-order kinetic profile revealed that the ZnO nanohybrid prepared from an inorganic–magnetic–organic composite had higher activity than that prepared from an inorganic–magnetic composite. This approach offers a viable plan for addressing energy and water purification issues using clean, non-harmful power sources.^108^

**Fig. 2:**
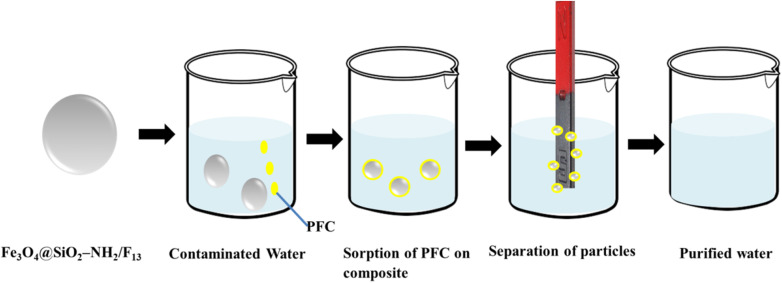
Removal of PFC particles from surface water using Fe_3_O_4_@SiO_2_–NH_2_/F_13_ magnetic hybrid composite. This figure has been reproduced from ref. 113 with permission from Elsevier, copyright 2016.

Composites of natural polymers have great potential as sorbents for magnetic solid-phase extraction (MSPE). Chitosan, a natural polymer known for its amino and carboxylic groups, offers biodegradability, biocompatibility, and ease of modification.^109–111^ Recently, magnetic chitosan nanohybrid materials were created by dispersing ferrites throughout the chitosan matrix, forming ternary ferrites chitosan microspheres (TFCM).^112^ The nanoparticles were prepared using a co-precipitation method, and the chitosan matrix was incorporated to produce an active adsorbent for the degradation of methylene blue dye. Zhou *et al.* prepared magnetic Fe_3_O_4_@SiO_2_–NH_2_/F_13_ hybrid material using a one-step reaction to modify the surface of SiO_2_ by an amino group and an octyl-perfluorinated chain through a sol–gel procedure. The resulting material (Fe_3_O_4_@SiO_2_–NH_2_/F_13_) exhibited tremendous adsorption ability of perfluorinated compounds (PFC) from the aqueous sample, as confirmed by HPLC-MS/MS analysis. 50 mg of the Fe_3_O_4_@SiO_2_–NH_2_/F_13_ sorbent in a 500 mL water sample reached the adsorption equilibrium within 30 minutes. Fluorine–fluorine interactions, electrostatic solid attraction, and size exclusion effects are attributed to the high adsorption efficiency. The recovery rates ranged from 90.65% to 106.67%, with low limits of detection (LODs) between 0.029 and 0.099 ng L^−1^. Therefore, Fe_3_O_4_@SiO_2_–NH_2_/F_13_ is an effective adsorbent for measuring the concentration of PFC in large volumes of water samples (Fig. 2).^113^

Qin *et al.* significantly synthesized MNPs, which were coated with mesoporous silica, which was modified using methyl dimethoxy and *p*-toluenesulfonic acid (PTSA) to prepare Fe_3_O_4_/mSiO_2_–Me–PTSA material. This material was tested for removing polychlorinated biphenyls (PCBs) from wastewater. The material facilitated rapid magnetic separation, with an adsorption time of just 10 minutes for PCBs. The Fe_3_O_4_/mSiO_2_–Me–PTSA showed high enrichment factors for PCBs (119 to 147) and an adsorption efficiency of 46.3 mg g^−1^. The process demonstrated promising outcomes with recoveries between 85.25% and 118.60% and low LODs ranging from 0.16 to 0.91 ng L^−1^.^114^ Wu *et al.* synthesized a polymorphic graphene-like porous carbon with N-doping/Ni magnetic nanohybrids. The NC@Ni-1 sample demonstrated exceptional long-term cycle stability, exceptional rate performance, and high specific capacity. The NC@Ni-0.75 sample showed a high adsorption capacity for MG dye (889 mg g^−1^). It also demonstrated a distinctive magnetic separation characteristic, making it suitable for the adsorptive removal of cationic MG dye.^115^

Choudhry *et al.* discuss synthesizing magnetic flax seeds (MFS), a unique hybrid nanocomposite based on iron oxide and flax seeds for water treatment. A straightforward co-precipitation technique was used to create the composite. With an equilibrium adsorption capacity of roughly 10 mg g^−1^ and the highest adsorption capacity (*Q*_max_) of 52.91 mg g^−1^, the experimental results revealed that MFS successfully absorbed over 90% of malachite green dye (MG) from a ten mg L^−1^ solution at 30 °C at neutral pH in 15 minutes. Weak electrostatic and hydrogen bonding interactions drove the adsorption process, which followed the Freundlich isotherm, indicating multilayer physisorption. The rate-determining phase was intraparticle diffusion. According to kinetic analyses, the pseudo-second-order model described MG adsorption. While MFS proved effective for water treatment, the study suggests further investigation of desorption and regeneration for future applications.^107^

Elanchezhiyan *et al.* effectively synthesized zirconium-imprinted manganese ferrite (ZrMnFe_2_O_4_@rGO) nanohybrids based on rGO and assessed their capacity to adsorb PFOA and PFOS. As validated by Raman spectroscopy, appropriate *I*_D_/*I*_G_ ratios demonstrated the intercalation of Zr–MnFe_2_O_4_ nanoparticles within rGO. Interactions with the rGO sheets decreased the Zr–MnFe_2_O_4_ nanohybrids' saturation magnetization. According to kinetic studies, the highest adsorption efficiency for PFOA and PFOS on Zr–MnFe_2_O_4_@rGO was achieved in less than 12 hours. The adsorption course was pH-dependent for both pollutants and adhered to a pseudo-second-order kinetic model. The Langmuir model, with maximum adsorption capacities of 10.1 mg g^−1^ for PFOA and 12.6 mg g^−1^ for PFOS, mainly attributable to electrostatic and hydrophobic interactions, was found to have the best fit to the experimental data, according to adsorption isotherm analysis. Notably, the adsorption density of Zr–MnFe_2_O_4_@rGO remained unchanged after four consecutive recycling tests, with no observable mass loss of the material.^116^

#### 3.1.2 Emerging contaminants

Wastewater comprises diverse and complex chemical contaminants; although around 700 have been identified, mainly harmful heavy metals, and numerous others, especially organic pollutants, remain unknown due to their diversity and low levels.^117^ Mostly ions of Pb, Ni, Cr, Cu, Co, As, Cd, Zn, and Hg, originate from mining, tanneries, fertilizers, painting, metal plating, and batteries.^118–121^ Heavy metals are not biodegradable and toxic; they persist in the aquatic system for extended periods in various oxidation states. Urban pollution also brings heavy metals from contaminated air, dust, and sediment, transportable commodities. These metals are most toxic in their biological forms, particularly to ecosystems and humans. Consequently, it is necessary to filter out these metals from the wastewater before they are released into water sources. Due to their large surface area and adequate adsorption capacities, magnetic nanohybrid materials such as magnetic activated carbon, magnetic metal–organic frameworks (MOFs), and magnetic mesoporous carbon are frequently employed to eliminate heavy metals from wastewater. Magnetic nanoparticles doped or deposited on biomass materials, such as chitosan-coated magnetic nanoparticles or magnetic carbon generated from coconut shells, can create biomass-based magnetic nanohybrid adsorbents that additionally showed efficacy in applications involving the removal of heavy metals.

#### 3.1.3 Removal of metal ions

Amin *et al.* aim to design affordable magnetite nanoparticles for the removal of heavy metals from water. MNPs were made using the thermal decomposition of iron oleate precursors, and the surface of these nanoparticles was covered with layers of mesoporous silica using cetyltrimethylammonium bromide. Chitosan coating was then applied to the coated particles. The adsorption study revealed that the adsorption was monolayer chemical adsorption, and the adsorption capacity was 150.33 mg g^−1^ for Pb^2+^ and 126.26 mg g^−1^ for Cd^2+^, which was entirely satisfactory.^122^ Creating a magnetic nanocomposite material, magnetic graphene oxide-covalently functionalized tryptophan, required functionalizing graphene oxide with tryptophan and adding magnetic characteristics to enhance its retrievability after use. The significant adsorption capacity of the material of 766.1 mg g^−1^ demonstrated its effectiveness in eliminating lead(ii) ions, indicating its potential for use in lead remediation. GO was used to eliminate cadmium(ii) ions, indicating a noteworthy adsorption capacity of 530 mg g^−1^, confirming its efficacy in eliminating cadmium. The high adsorption capabilities attained imply that the materials worked well in the experimental setup of the study.^123^

Muntean *et al.* synthesized carbon magnetic nanocomposite and used it as an adsorbent to extract heavy metal ions from aqueous solutions, including copper, lead, and zinc. Working at the natural pH of 5.8 for 240 minutes and utilizing a dose of 1 g L^−1^ of the nanocomposite were the ideal circumstances for the process, which produced high removal efficiencies of 81.36% for copper, 84.50% for lead and 72.68% for zinc ions. The investigation results showed that these parameters were appropriate for effective heavy metal ion removal operations employing the carbon magnetic nanocomposite material since they struck a good balance within removal rate, practicability, and adherence to ecological and socioeconomic issues.^124^ The chitosan matrix can promote the growth of MNPs and, at the same time, hinder their aggregation. Tolessa *et al.* synthesized two μm magnetic chitosan hybrid materials through suspension cross-linking to eliminate Ag NPs. They suspended MNPs in 1% chitosan solution and added toluene. The mixture was stirred at 500 rpm for half an hour; then, the solutions of NaOH and glutaraldehyde were added. The magnetic chitosan composite material was separated from the solution with the help of an external magnet. Alongside inductively coupled plasma-mass spectrometry (ICP-MS), the material provided low LODs for Ag NPs, from 0.016 to 0.023 μg L^−1^. The high removal efficiency of Ag nanoparticles, between 84.9% and 98.8%, is due to the positive charges on the chitosan's surface, making it an excellent adsorbent. The magnetic chitosan hybrid material remains effective after three recycling uses, with an efficiency of approximately 77.2 ± 2.2%.^125^

In another study, a core–shell Fe_3_O_4_@RF@mTiO_2_ material was developed and tested as an MSPE sorbent for removing arsenic from highly acidic samples by Zhao *et al.* The material featured a 130 nm Fe_3_O_4_ core, a 50 nm resorcinol–formaldehyde (RF), and a mesoporous TiO_2_ shell. This design provided quick adsorption, which is 1.16 g mg^−1^ h^−1^ and a 139 mg g^−1^ adsorption capacity. The RF layer offered hydrophobic protection to the Fe_3_O_4_ core against acid etching, while the mesoporous TiO_2_ enabled surface complexation and electrostatic interactions with arsenate. The magnetic properties allowed facile separation and recycling using an external magnetic field. This multi-layer material shows promise for long-term wastewater treatment.^126^ The adsorption capabilities of magnetic iron oxide nanoparticles for heavy metals, including Pb, Cd, Cu, Zn, and Co, in wastewater treatment procedures are significantly increased by modification with chemicals such as thiosalicylhydrazide. Hybrid magnetic nanohybrid materials have been synthesized to overcome the limits of original magnetic nanoparticles in water remediation and to advance heavy metal removal efficiency by combining magnetic capabilities with other compositions. Magnetic nanohybrid materials with enhanced adsorption capabilities and practicality for environmental remediation applications have been developed due to hybridization operations' new compositions and chemical structure alterations.^127^

**Fig. 3:**
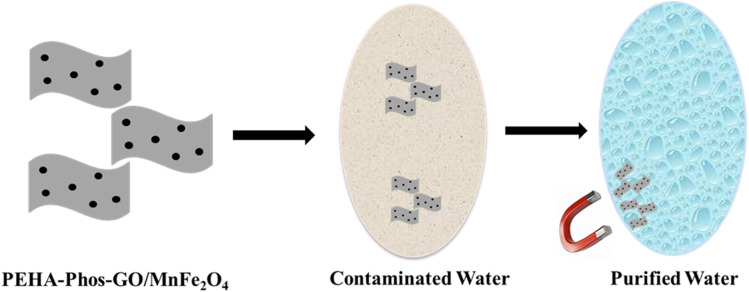
Removal of lead ions using PEHA–Phos–GO/MnFe_2_O_4_ nanohybrid.

A flower-like magnetic MoS_2_/Fe_2_O_4_ nanohybrid has been successfully synthesized by Wang *et al.* and used for the removal of Pb(ii) and Hg(ii) from contaminated wastewater and soil. The nanohybrid was highly efficient in removing heavy metal ions, with rates of 99.56% for Pb(ii) and 99.08% for Hg(ii). PH, contact time, initial metal ion concentration, and temperature significantly influence the removal efficiency. The nanohybrid also demonstrated excellent reusability after multiple adsorption and desorption cycles, indicating its economic and environmental sustainability. The study concludes that the flower-like magnetic MoS_2_ nanohybrid is a promising material for efficient remediation of these contaminants, with its high removal efficiency, reusability, and sensitivity to various parameters making it a valuable tool for environmental cleanup processes.^128^ Using a facile approach, Dai *et al.* synthesize a ternary adsorbent, PEHA–Phos–GO/MnFe_2_O_4_. This material showed improved Pb(ii) adsorption properties compared to GO because of the large number of adsorption sites offered by the amino and phosphate groups. Experimental data showed that the adsorption process depended on the pH and involved pseudo-second-order, which pointed to chemisorption as the rate-controlling step. The data obtained from the Langmuir isotherm agreed with the experimental data. The maximum adsorption capacity for Pb(ii) was 366.4 mg g^−1^ at pH 5.5, which was higher than that of pristine GO, 212.1 mg g^−1^. Recycling tests further supported the higher capacity of regeneration of PEHA–Phos–GO/MnFe_2_O_4_, and the work demonstrated the possibility of applying the synthesized material in water treatment processes, as shown in Fig. 3.^129^

**Fig. 4:**
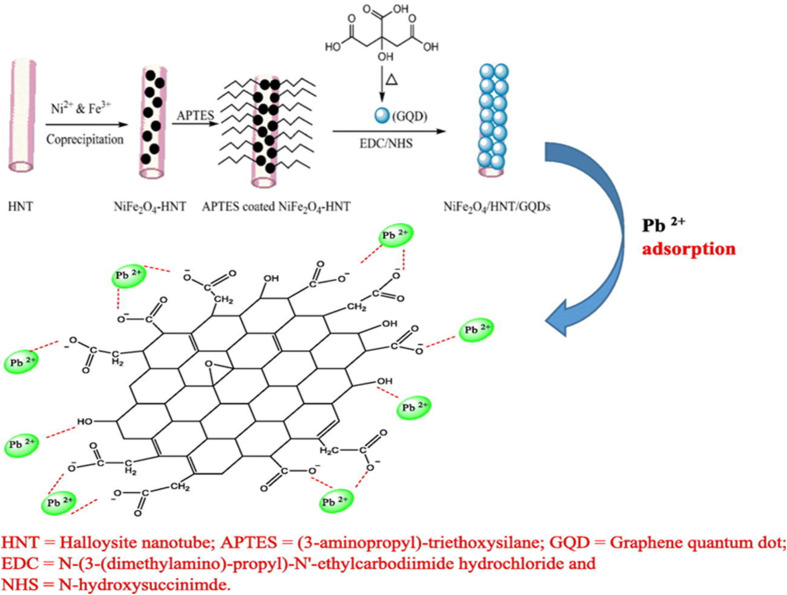
Synthesis of NiFe_2_O_4_/HNTs/GQDs and plausible adsorption mechanism of Pb(ii) ions onto NiFe_2_O_4_/HNTs/GQDs. This figure has been adapted from ref. 130 with permission from Elsevier, copyright 2020.

Pirhaji *et al.* successfully synthesized nano NiFe_2_O_4_/HNTs/GQDs as a novel adsorbent for Pb(ii) removal (Fig. 4). Batch experiments revealed that its strong adsorption performance is mainly due to complexation interactions between Pb^2+^ ions and the functional groups on the adsorbent. The Box–Behnken design and response surface methodology determined optimal experimental parameters, achieving a maximum Pb removal efficacy of 97.14%. The Langmuir isotherm model best explained the adsorption behavior, with a *Q*_max_ of 42.02 mg g^−1^ at 25 °C. The Elovich model suggested chemical adsorption and kinetic studies indicated that the process followed a pseudo-second-order model. The thermodynamic analysis confirmed that the adsorption is endothermic and spontaneous, making NiFe_2_O_4_/HNTs/GQDs a cost-effective and efficient option for Pb(ii) removal.^130^

A new magnetic solid-phase extraction (MSPE) approach using Ni–Al layered double hydroxide/magnetite (Ni–Al LDH/Fe_3_O_4_) nano sorbent was synthesized by Abdolmohammad-Zadeh *et al.* for the enrichment and separation of Mn(vii)/Mn(ii) in water samples by flame atomic absorption spectrometry. The nanosorbent selectively adsorbs Mn(vii) because of the electrostatic attraction and intercalation of MnO_4_^−^ ions into the LDH layers. Mn(ii) is quantified indirectly by calculating the difference between the total Mn and Mn(vii) concentrations. The nano-hybrid is paramagnetic, allowing simple separation using an external magnet without filtration or centrifugation. The nanosorbent is non-toxic, recyclable more than 100 times, and offers high sensitivity, accuracy, and selectivity for Mn speciation in water samples.^131^

In another study, Katubi *et al.* focused on synthesizing the MnFe_2_O_4_/GO nanocomposite using an *in situ* method for the adsorption of Pb^2+^ ions in water. The adsorption parameters, including initial concentration, adsorbent dose, pH, and reaction time, were determined to identify the best condition for adsorption. The study revealed that with the increase in the initial concentration of Pb^2+^ ions, the adsorption capacity ranged between 63 and 625 mg g^−1^. Also, the extent of removal increased with the increase in the adsorbent dose from 39% to 98.8% as the number of active sites increased. The results also revealed that the adsorption process was most effective at pH 6. The adsorption capacity increased sharply during the first 15 minutes and then progressively at a slower rate as the surface became fully occupied, the maximum contact time being 120 minutes. The study also exhibited that Pb^2+^ adsorption fitted the Langmuir isotherm model, which supports monolayer adsorption with a maximum 636.94 mg g^−1^ adsorption capacity. Moreover, reusability tests proved the stability and efficiency of the nanocomposite for at least five cycles, thus making it an efficient and cost-effective adsorbent for Pb^2+^ ions in water treatment.^132^ rGO was synthesized utilizing the modified Hummers' method, and ZnFe_2_O_4_ nanoparticles were incorporated onto the surface of rGO to fabricate the adsorbent material to remove lead ions. Experimental parameters, including the amount of the adsorbent, initial concentration of the pollutant, and pH, were thus adjusted to maximize the pollutant removal efficiency. The developed rGO nanocomposite had an adsorption capacity of 89.8 mg g^−1^ for lead ions. Vacancy defects and oxygen-containing functional groups on the rGO surface were identified as the primary mode for pollutant elimination. Adsorption was consistent with the Langmuir isotherm and fitted a pseudo-second-order kinetic model, suggesting that the adsorption process was chemisorptive and through pollutant monolayers. The synthesized rGO nanocomposite was stable and reusable for up to five cycles, which is suitable for application in heavy metals and dye removal in the water treatment process.^133^

In another work, coprecipitation and Hummer's modified method were combined to synthesize the magnetic Fe_3_O_4_–GO nanohybrid composite material. The morphology of the hybrid sample demonstrated that the diffusion of Fe_3_O_4_ nanoparticles into the mesoporous GO layers of porous channels limited GO nanosheet restacking and stopped magnetic nanoparticle leaching and agglomeration. The pseudo-second-order kinetic and Langmuir isotherm models suit the results of the kinetic and isotherm analyses conducted to assess the adsorption mechanism quite well. After 60 minutes, the As(v) adsorption efficiency, H, peaked at 99.37%. The GO/Fe_3_O_4_ nanohybrid material in an acidic media showed a *Q*_max_ of 14.1 mg g^−1^. Further research was also done on the function and contribution of GO and Fe_3_O_4_ nanoparticles in adsorptive capacity and the enhancement of As(v) adsorption efficiency.^134^

### 3.2 MNPs for the cleanup of oil spills

NH_2_-functionalized magnetic materials can be used for extracting oil droplets from water.^135^ High magnetization and a large surface area are required for nanocomposites to have a strong oil absorption capability.^136^ Atta *et al.*^137^ synthesized a novel class of magnetic sorbents by employing the co-precipitation approach to coat magnetite nanoparticles with rosin amidoximes, which produced a hydrophobic coating on the surface of Fe_2_O_3_ nanoparticles. This hydrophobic coating can increase the oil uptake selectivity, stop Fe_2_O_3_ nanoparticles from oxidizing, and prevent magnetic nanoparticles from aggregating. By comparing iron oxide coated with RK-AN amidoxime to that coated with R-AN and bare Fe_3_O_4_ nanoparticles, oil recovery measurements indicate that the latter is a worse oil sorbent. Fe_3_O_4_ nanoparticles coated with RK-AN amidoxime exhibit a higher level of oil removal than those coated with R-AN amidoxime, as shown by their respective magnetization saturation values of 60.9 and 70.8 emu g^−1^.

Using the unique physicochemical characteristics of ZnO-T, PDMS, and Fe_2_O_3_-NR, a magnetic nanosorbent was effectively synthesized by Sharma *et al.* in 2019. With a WCA of 157°, the as-synthesized PZF nanosorbent exhibited superhydrophobic super oleophilic properties. With an oil removal (%) of 96% and an adsorption capacity of 1135 mg g^−1^, respectively, this magnetic nanosorbent efficiently separates diesel oil from water. The kinetics of oil adsorption showed that diesel oil adsorptive capacity on PZF nanosorbent is high, with equilibrium potentially reached in as little as 50 minutes. The pseudo-first-order kinetics and Langmuir isotherm models matched the oil adsorption process well. The simple synthesis process and the superhydrophobic–superoleophobic and magnetic responsive characteristics of the as-prepared nano sorbent offer an alternate method for treating oily wastewater.^138^ Shao and Yu successfully made a sepiolite/graphene oxide (Sep/GO) membrane for wastewater treatment. The excellent oil/water selectivity and low oil adherence of the synthetic Sep/GO membranes made them highly successful in separating oil-in-water emulsions. Moreover, the membrane preserved its underwater high stability and superoleophobicity in severe settings. As a result, the Sep/GO membrane works well in processes involving wastewater treatment and oil/water separation.^139^

Qian and Chen described a TiO_2_/sulfonated GO/Ag NPs membrane with beneficial photocatalytic and wettability capabilities.^140^ When solubilized methylene blue (MB) was exposed to UV light, the synthesized membrane demonstrated good photodegradation and an effective oil/water separation performance. As a result, the membrane has interesting uses in wastewater treatment and oil/water separation.^140^ Zhang *et al.* synthesized a superoleophilic and oil–superhydrophobic composite nanofibrous membrane (GPNM) for gravity-driven surfactant-stabilized water-in-oil emulsions using a straightforward one-step electrospinning technique. The synthetic composite membrane demonstrated a remarkable separation efficiency of 99.8% for water-in-oil emulsions. These findings revealed that the composite nanofibrous membrane could potentially treat oil-contaminated wastewater.^141^

In another study, Cheng and Barras effectively created a superoleophilic/superhydrophobic rGO–polydopamine membrane functionalized with 1*H*,1*H*,2*H*, and 2*H*-perfluorodecanethiol (rGO–PDA–PFDT) using a straightforward two-step procedure. The rGO–PDA–PFDT membrane was utilized to clean water by separating oil. The membrane's superhydrophobicity and superoleophilicity allowed it to extract chloroform from water. Therefore, in oil/water separation operations, the synthesized rGO–PDA–PFDT membrane is exceptionally favorable.^142^ Shokry *et al.* synthesized a novel magnetic sorbent material from water hyacinth to remove oil from polluted water. The chemical and thermal treatment established that the root segment of the water hyacinth had a higher oil adsorption capacity than the shoot segment. The comparison of the two segments with nano-activated carbon and NMAC (nano-magnetic activated carbon) showed that NMAC had a more crystalline structure, better pore structure, and thermal stability. It also registered a saturation magnetization of 17.2587 emu g^−1^. Oil removal efficiency increased with the solution temperature and NMAC dosage increase. The results indicated that the Freundlich isotherm model best described the oil adsorption process rather than the Langmuir isotherm and Dubinin–Radushkevich models, implying monolayer adsorption and surface heterogeneity. The pseudo-second-order and intra-particle diffusion models were utilized to determine adsorption kinetics. The primary process through which oil was adsorbed on NMAC was physical absorption through van der Waals forces.^143^

Sharma *et al.* investigated the physicochemical characteristics of ZnO-T, PDMS, and Fe_2_O_3_-NR to give large surface area, low surface energy, and super-paramagnetism, respectively, in synthesizing a magnetic nanosorbent. The PZF nanosorbent exhibited superhydrophobic and superoleophilic properties, exhibiting a WCA 157°. With an oil removal percentage of 96% and an adsorption capacity of 1135 mg g^−1^, respectively, this magnetic nanosorbent efficiently separates diesel oil from water. According to the oil adsorption kinetics, diesel oil can quickly adsorb on PZF nanosorbent, reaching equilibrium in as little as 50 minutes. The Langmuir isotherm and pseudo-first-order kinetics models fit the oil adsorption process well. The easily synthesized nanosorbent's superhydrophobic and superoleophilic properties, combined with its magnetic responsiveness, offer a novel approach to treating oily wastewater.^138^

### 3.3 MNPs for photocatalytic degradation of organic dyes

**Fig. 5:**
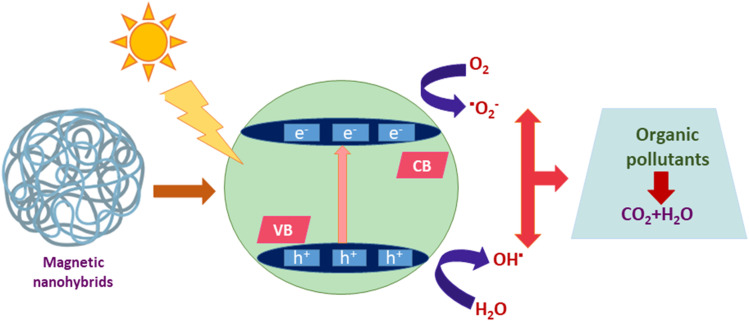
General mechanism for organic dye degradation by photocatalysis.

Among the various pollutants, waterway dyes are recognized as a global ecological concern that can impact humans, plants, and animals. Materials that are widely studied for the photocatalytic degradation of organic dyes found in water effluents are magnetic nanohybrids. Organic dye degradation *via* photocatalysis using magnetic nanohybrid materials follows the mechanism shown in Fig. 5.

Yuan *et al.* prepared Fe_3_O_4_@SiO_2_@meso-TiO_2_ nanocomposites with a core–shell architecture by a simple and efficient method with increased photocatalytic performance. The magnetic core, SiO_2_ interlayer, and mesoporous TiO_2_ outer shell constitute the as-made core–shell architecture. It was produced with TiOSO_4_ as the titanium source and cetyltrimethylammonium bromide (CTAB) as a pore-forming agent. Fe_3_O_4_@SiO_2_ was made using a sol–gel method. In comparison to Fe_3_O_4_@SiO_2_@solid-TiO_2_, the Fe_3_O_4_@SiO_2_@meso-TiO_2_ composite showed better photocatalytic efficiency for the degradation of Rh B in aqueous suspension due to the large BET surface area produced by the mesoporous TiO_2_ shell.^144^ Aliyan *et al.* have explained the immobilization of Fe_3_O_4_ to modify the surface of meso-structured silica (SBA-15) to produce a nanocatalyst. Aliyan *et al.* used a UV lamp as a light source to investigate the catalytic activity of Fe_3_O_4_@SBA-15 toward the photodegradation of MG in a photo-catalytic reactor. The impact of several experimental factors on the process's degrading performance was assessed by analyzing catalyst dose, initial dye concentration, and dye solution pH in the presence of Fe_3_O_4_@SBA-15 as a photo-catalyst. Even after five MG degradation cycles, the described photocatalyst showed notably high catalytic stability and low activity loss.^145^ Sahoo *et al.* investigated the adsorption of methyl orange (MO) on monodispersed magnetic mesoporous manganese ferrite composites with a size of around 200 nm. Sonication and photolysis in the presence of sunshine improved the MO's degrading efficiency. The authors concluded that their catalyst exhibited adsorption, degradation, recovery, and reusability in a single system. The electron transfer activities inside the MnFe_2_O_4_ particles cause the potential dye degradation reaction. The following describes the potential method by which Mn^2+^ ions decompose H_2_O_2_.^146^ Yonghong Ni *et al.* found that adding Mn^2+^ to TiO_2_ nanoparticles increased their photocatalytic activity and that the metal ions, such as Fe^2+^, actively contributed to the degradation of H_2_O_2_ to produce OH radical species.^147^Mn^2+^ + H_2_O_2_ → Mn^3+^ + HO˙ + OH^−^H_2_O_2_ + HO˙ → H_2_O + HOO˙Mn^3+^ + HOO˙ → Mn^2+^ + H^+^ + O_2_Fe^3+^ + HOO˙ → Fe^2+^ + O_2_ + H^+^

The organic dye (RH) reacts with the O_2_ molecule and OH produced above.R–H + HOO˙ → X + H_2_O + CO_2_X = degraded organic product.

Santos *et al.* prepared the magnetic CoFe_2_O_4_/TiO_2_ composite through a facile solvothermal route. Compared with pristine TiO_2_, the CoFe_2_O_4_/TiO_2_ catalyst had a reduced band gap, allowing it to exhibit higher photocatalytic efficiency. The nanohybrid catalyst showed exceptional activity in degrading Erionyl Red A-3G dye, with 69.4% TOC eradication after 120 minutes and 95.39% color removal after 15 minutes of reaction. One possible explanation for the degradation of Erionyl Red A-3G is that when the CoFe_2_O_4_/TiO_2_ composite is exposed to UV light, the TiO_2_ in the composite absorbs light and produces more charges. As a result, holes (h^+^) are formed on the valence band (VB) of TiO_2_ due to an electron transfer (e^−^) from VB to CB. A successful e^−^/h^+^ pair separation is subsequently promoted by the photogenerated electrons (e^−^) migrating from the CB of TiO_2_ to that of CoFe_2_O_4_, which helps to limit photogenerated charge recombination and enhance photodegradation activity. The e^−^ on the CB of CoFe_2_O_4_ reacts with O_2_ to produce O_2_˙^−^ radical, which destroys pollutants. While the h^+^ on the VB of TiO_2_ directly oxidizes organic molecules and combines with OH^−^ or H_2_O to produce ˙OH radical, which also functions as an oxidizing species.^148^TiO_2_ + *hν* → e^−^ + h^+^CoFe_2_O_4_ + TiO_2_ → CoFe_2_O_4_ + TiO_2_ (e^−^)e^−^ + O_2_ → O_2_˙^−^O_2_˙^−^ + Erionyl dye → degradation products
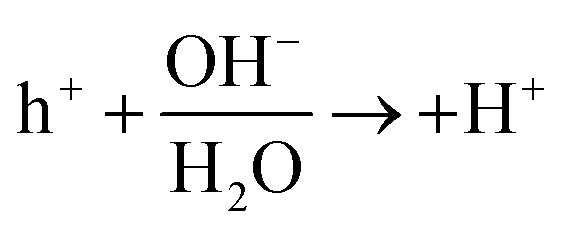
OH˙ + Erionyl dye → degradation product

**Fig. 6:**
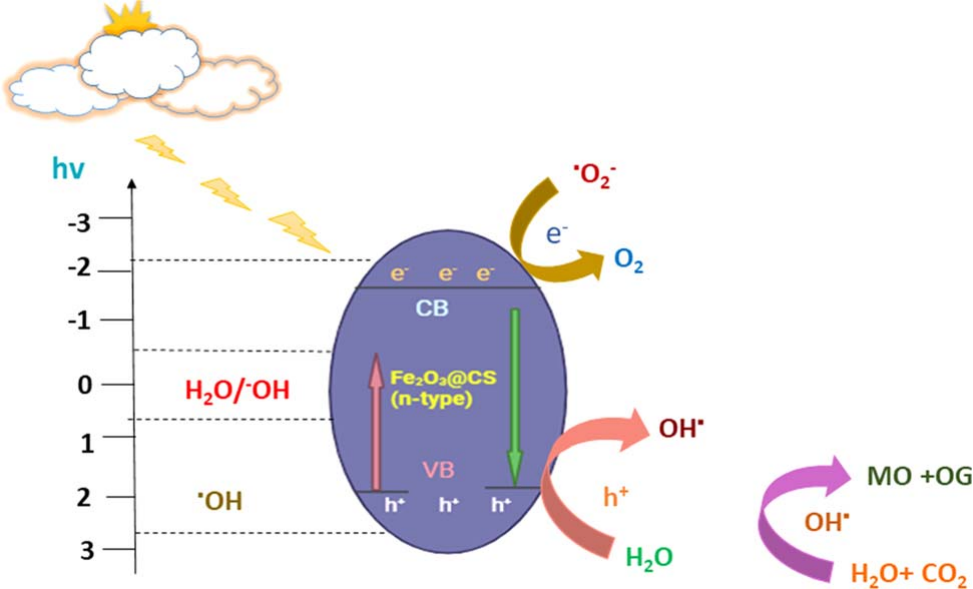
Illustration of MO and OG dye molecule oxidation after e^−^–h^+^ production on a Fe_2_O_3_@CS photocatalyst.

Rehman *et al.* synthesized Fe_3_O_4_@GO by modifying 3-aminopropyl triethoxysilane (APTES) and tetraethyl orthosilicate (TEOS) using a mechanical stirring technique. Under UV light, the synthesized nanocomposite was evaluated as a possible heterogeneous catalyst for methylene blue (MB) degradation. Over time, the catalysts' photocatalytic activity increased progressively. The Fe_3_O_4_@GO composite catalyst had the highest MB removal efficiency of 70.06%, which was more excellent than pure Fe_3_O_4_ (57.56%).^149^ Vigneshwaran *et al.* reported that co-precipitation created the Fe_2_O_3_-reinforced chitosan (Fe_2_O_3_@CS) nanocomposite. The surface of the chitosan was made available to develop a beneficial nanocomposite. Because of the increased partition efficiency and quicker transfer of the photo-generated charge carriers, these Fe_2_O_3_ wrappings on chitosan offer synergistically enhanced characteristics. MO and OG dyes were degraded using this combination. According to the data, the most significant degradations for MO and OG were 89.2% and 94.6%, respectively. The trapping study highlighted how OH radicals contributed to dye degradation over Fe_2_O_3_@CS composites. The suggested method used Fe_2_O_3_@CS to oxidize anionic MO and OG dyes, as shown in Fig. 6.Fe_2_O_3_@CS → *hν* (h^+^ + e^−^) Fe_2_O_3_@CS(e^−^ + h^+^) → O_2_˙^−^ + OH˙O_2_˙^−^ + H^+^ → OH˙OH˙ + OH˙^−^ → H_2_O_2_H_2_O_2_ + e^−^ → OH˙ + OH^−^h^+^ + H_2_O → OH˙ + MO + OG → by-products + HO_2_ + CO_2_

Furthermore, the production of reactive species increases quickly due to the e^−^–h^+^ pair's delayed recombination. It has a significant impact on the materials' photocatalytic efficiency.^150^

**Fig. 7:**
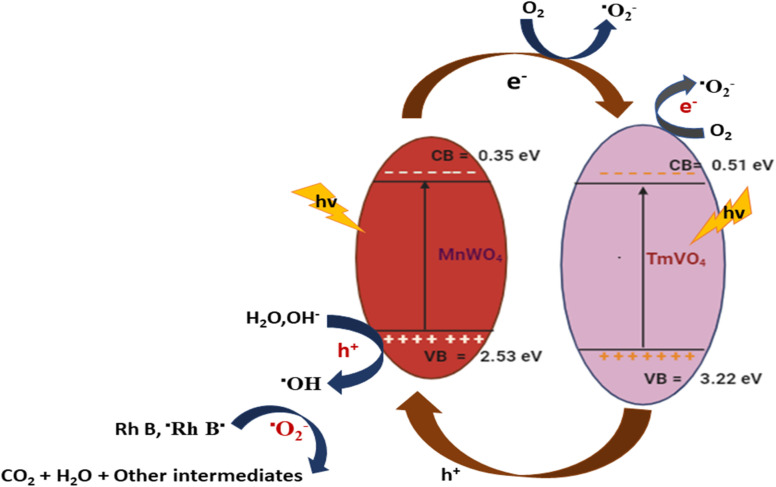
Rh B degradation scheme in the presence of ternary nanohybrids.

Sobhani-Nasab *et al.* successfully created a novel MnWO_4_/TmVO_4_ ternary nanohybrid using a straightforward sonochemical technique. Under visible light, the degradation of phenol red (Ph R), eosin Y (EY), 2-naphthol, and rhodamine B (Rh B) by MnWO_4_/TmVO_4_ was examined. The corresponding photocatalytic efficiencies for Rh B, Na, Ph R, and EY are 99.2%, 93.3%, 88.3%, and 83.1%.^151^ When MnWO_4_/TmVO_4_ is excited by visible light, the photogenerated holes on the VB of TmVO_4_ will move to MnWO_4_, while the photoinduced electrons on the CB of MnWO_4_ will migrate to TmVO_4_ (depicted in Fig. 7). The heterojunction forms relatively tiny amounts of 
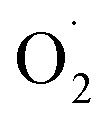
 and OH˙. The degradation results demonstrate that MnWO_4_/TmVO_4_ has a greater photodegradation capacity than pure TmVO_4_ or MnWO_4_.^152^

Roostaee *et al.* investigated the reduction process by sodium citrate, Hummer's method, and hydrothermal approach was used to synthesize Fe_3_O_4_@Au core–shell nanoparticles decorated on rGO nanocomposite, respectively. These nanostructures were used to break down crystal violet (CV) by photocatalysis. Under ideal circumstances and visible light irradiation, it was found that the Fe_3_O_4_@Au–rGO performed better than Fe_3_O_4_@Au and rGO alone. In the presence of 0.008 g of the produced nano-photocatalyst, 100% degradation was obtained after 1 minute, and in the presence of 0.005 g, 100% degradation was obtained after 5 minutes.^153^ Because rGO can function as an effective electron transporter for separating photo-generated electron–hole pairs through interfacial charge transfer, Fe_3_O_4_@Au–rGO demonstrated remarkable photocatalytic activity to destroy crystal violet.^154^

### 3.4 MNPs for photocatalytic degradation of emerging contaminants

**Fig. 8:**
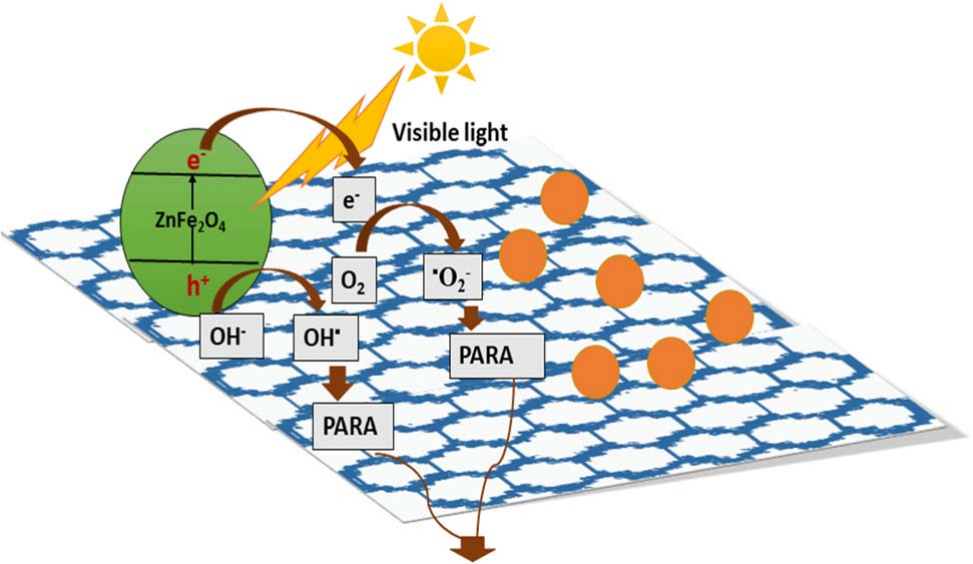
A graphical representation of PARA's photocatalytic degradation over the ZnFe_2_O_4_–*x* wt% GR nano-hybrid.

Emerging contaminants (EC) are new types of pollutants recently introduced into aquatic systems that harm the environment and the health of living things. Personal care products (PPCPs) and medications are prominent examples of emerging pollutants.^155^ Since PPCPs are more difficult to degrade than other organic contaminants, magnetic nanohybrids are an excellent option for eradication.^156^ Using the sol–gel technique, Rostami *et al.* synthesized ZnFe_2_O_4_–4 weight percent GR nano-hybrids. It is evident that when exposed to visible light, ZnFe_2_O_4_ itself is nearly photo-catalytically inert; however, the addition of graphene significantly improves photocatalytic activity. Under exposure to visible light, the ZnFe_2_O_4_–*x* weight percent GR nano-hybrids demonstrated enhanced charge separation and nano-photocatalytic activity towards PARA decomposition. Fig. 8 is a potential mechanism for increasing nano-photocatalytic activity.^157^ZnFe_2_O_4_ + *hν* → ZnFe_2_O_4_ (h^+^ + e^−^)ZnFe_2_O_4_ (e^−^) + graphene → ZnFe_2_O_4_ + graphene (e^−^)Graphene (e^−^) + O_2_ → O_2_˙^−^ + grapheneZnFe_2_O_4_ (h^+^) + OH^−^ → ZnFe_2_O_4_ + OH˙ZnFe_2_O_4_ (h^+^) + OH˙ + O_2_˙^−^ + PARA → intermediate products + CO_2_ + H_2_O

Tang *et al.*, using a hydrothermal process, prepared rGO–CdS/ZnS heterostructure composites that have been effectively synthesized by building the CdS/ZnS heterostructure nanoparticles on rGO sheets. In contrast, GO is reduced at the same time. With 15% RGO content, the rGO–CdS/ZnS composites, as synthesized, show very active TC photodegradation (about 85%). When RGO is added, the rGO–CdS/ZnS heterostructure photocatalysts show less electron–hole pair recombination than CdS/ZnS, facilitating better charge-carrier migration and photocatalytic activity.^158^ The charge transfer and photocatalytic activity by RGO–CdS/ZnS composites are schematically illustrated in figure. The production of holes in the VB is caused by the very initial excitation of the electron–hole pairs in CdS, which stimulates the electrons to the conduction band (CB). Because of the band potential, the electrons in the CB of CdS may be immediately transported into the CB of ZnS. The potential of ZnS is around 0.91 eV, which is less negative than the CB level of CdS (about 0.95 V). Additionally, RGO may trap the free photo-generated electrons in the ZnS CB, which are linked to the 2D carbon network. Thus, the visible-light activity was significantly produced by the photo-generated electrons of ZnS and RGO being so active that they participated in the surface reaction to make radicals.^159^

**Fig. 9:**
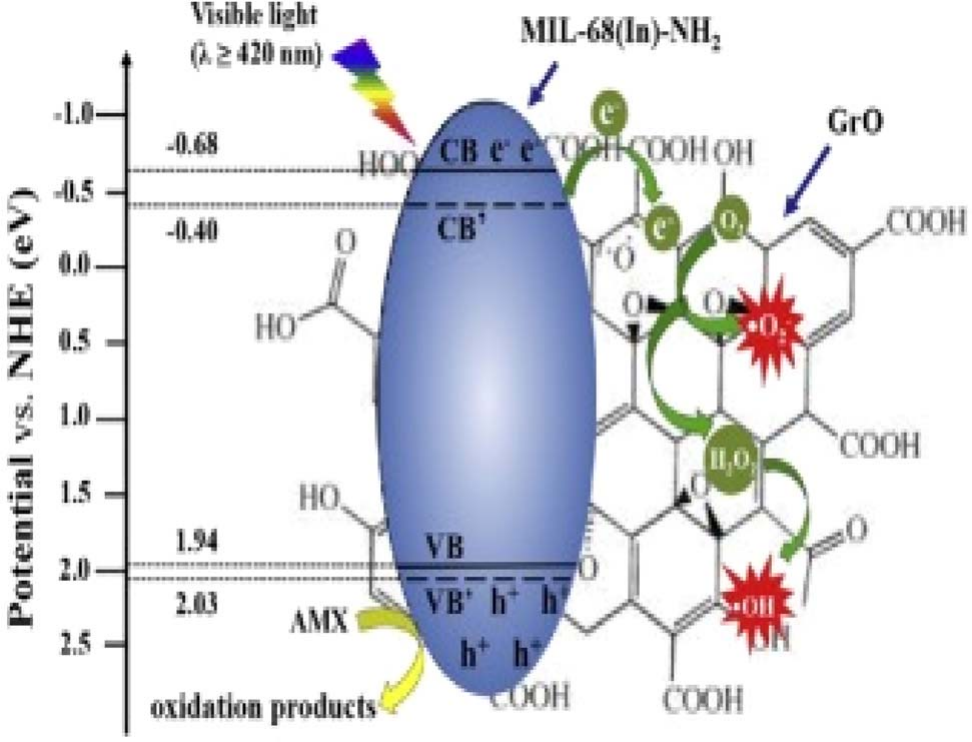
Photocatalytic mechanism of MIL-68(In)-NH_2_/GO composite. This figure has been adapted from ref. 160 with permission from Elsevier, copyright 2017.

To degrade amoxicillin (AMX), a new MIL-68(In)-NH_2_/GO composite was prepared as a visible-light-driven photocatalyst. Compared to pure MIL-68(In)-NH_2_, the MIL-68(In)-NH_2_/GO composite demonstrated much greater photocatalytic activity. After 120 and 210 minutes of irradiation, respectively, 93% degradation and 80% TOC removal toward AMX were possible using 0.6 g per L MIL-68(In)-NH_2_/GO and pH = 5. The modification by employing GO, which worked as a sensitizer to increase visible light absorption in addition to acting as an electron transporter to reduce photogenerated carrier recombination, is responsible for the increased activity for MIL-68(In)-NH_2_/GO.^160^ The photocatalytic mechanism for the degradation of AMX is shown in Fig. 9.

**Fig. 10:**
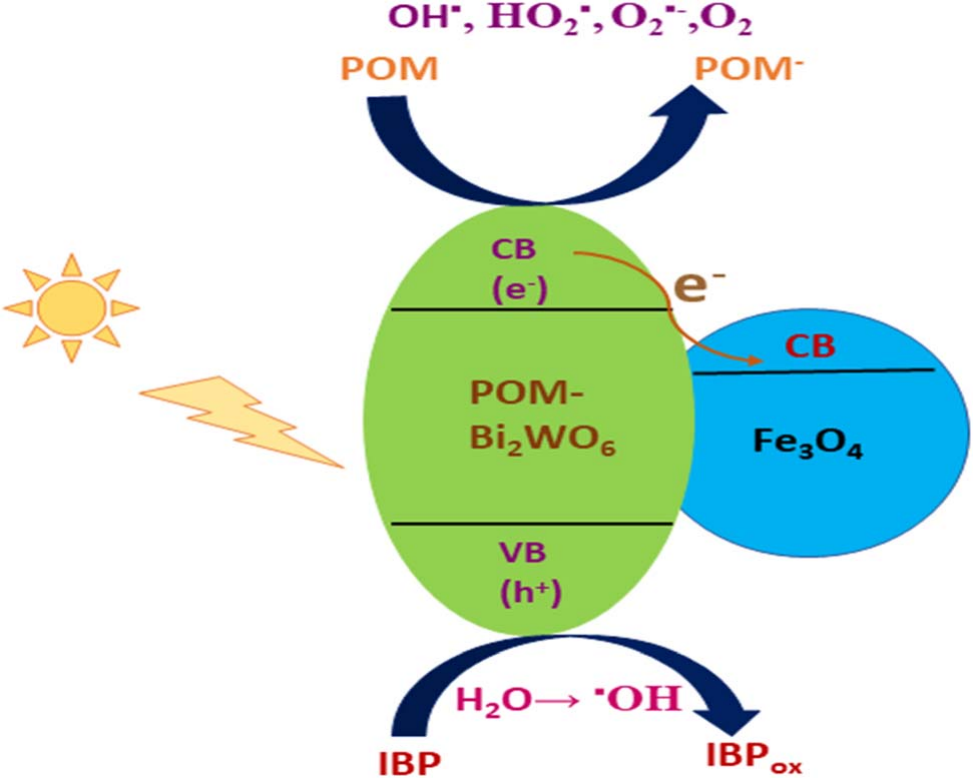
A schematic illustration of the IBP photocatalytic reaction on the Fe_3_O_4_/Bi_2_WO_6_ nanohybrid.

MIL-68(In)-NH_2_/GO's ECB and EVB have been estimated to be −0.40 and 2.03 eV *vs.* NHE, respectively. The photogenerated electrons on the CB of MIL-68(In)-NH_2_ are beneficial for reducing the O_2_ because the ECB of MIL-68(In)-NH_2_/GO (−0.40 eV) is more damaging than the O_2_/˙O_2_^−^ potential (−0.046 eV *vs.* NHE).^161^ Similarly, these electrons can also reduce O_2_ to H_2_O_2_ (O_2_/H_2_O_2_ is 0.915 eV *vs.* NHE), and by trapping one electron, the resulting H_2_O_2_ can be further converted into ˙OH. Due to the lower positive potential of MIL-68(In)-NH_2_/GO's VB (2.03 eV) compared to the typical ˙OH/OH^−^ (2.38 eV *vs.* NHE), the photogenerated holes on the VB of MIL-68(In)-NH_2_/GO can simultaneously oxidize the AMX directly, but not the OH^−^ to form ˙OH.^162^ Bastami *et al.* reported that magnetic Fe_3_O_4_/Bi_2_WO_6_ nanohybrids were created in two steps. A solvothermal technique was used to develop Fe_3_O_4_ nanospheres in a polyol medium, and a subsequent hydrothermal process produced Bi_2_WO_6_ nanocrystals. Tungstophosphoric acid hydrate (H_3_PW_12_O_40_) was used as an acidic agent in the Bi_2_WO_6_ nanoparticle manufacturing. Ibuprofen (IBP) was photo-catalytically degraded from an aqueous solution under sunlight to assess the photocatalyst's activity. Particularly at pH 4.7, the as-prepared nanohybrid demonstrated significant efficiency in the photocatalytic destruction of IBP and was readily recovered by a magnet.^163^ The OH˙ radical may be produced due to a chain reaction when hydrogen peroxide comes into contact with Fe(iii) sites on the surface of the Fe_3_O_4_/Bi_2_WO_6_ photocatalyst.^164^ First, the photocatalyst surface produces the Fe(iii)H_2_O_2_ complex. Then, the generated species is transformed into Fe(ii) and 
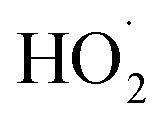
. Additionally, Fe(ii) species are produced on the photocatalyst surface. Every type of Fe(ii) species produced on the photocatalyst surface has the potential to react with H_2_O_2_ and create reactive radicals (
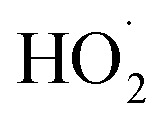
 and HO˙) (Fig. 10).

**Fig. 11:**
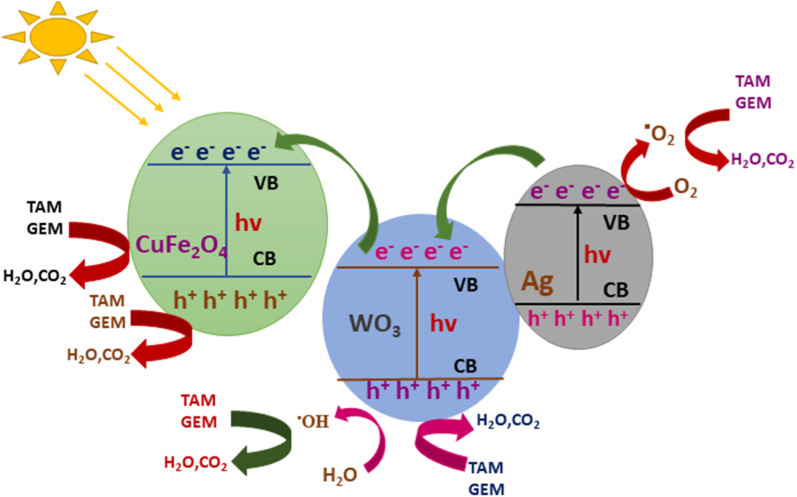
Photodegradation mechanism of the Ag–CuFe_2_O_4_@WO_3_ nanoparticles in the drug models.

The Fe_3_O_4_/Bi_2_WO_6_ photocatalyst's polyoxometalate-based materials (POM) are considered to increase photo-efficiency by inhibiting the rapid recombination of a charge pair (h^+^–e^−^) on nanohybrids and generating a potent oxidant 
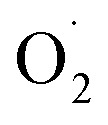
.^165^ When sunlight irradiation excites Fe_3_O_4_/Bi_2_WO_6_, the electron shifts from its conducting band to the POM's LUMO; following the attack of radicals on organic molecules, the adsorbed O_2_ and H_2_O_2_ can readily trap an electron in the LUMO of the POM anion to produce the oxidizing species OH˙. Consequently, the enhanced photocatalytic activity can slow down the rapid recombination of photo-induced electrons and holes. Sayadi *et al.* produced a novel Ag–CuFe_2_O_4_@WO_3_ magnetic photocatalytic nanosystem and examined how well it degraded two drug pollutants, gemfibrozil (GEM) and tamoxifen (TAM), when exposed to UV light. At pH 5, a photocatalyst dosage of 0.2 g L^−1^, a drug concentration of 5 mg L^−1^, and a contact time of 150 minutes, the highest levels of photodegradation for GEM (81%) and TAM (83%) were attained. Excellent catalytic efficiencies were produced by the slow electron–hole recombination brought about by the photocatalyst's heterostructure. The following reactions can be used to characterize the primary mechanism of the drug models' photodegradation process, and the mechanism can be seen in Fig. 11.^166^
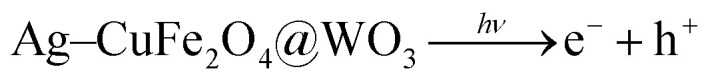
O_2_ +e^−^ → O_2_˙^−^O_2_˙^−^ + 2H^+^ → H_2_O_2_OH^−^ + h^+^ → OH˙H_2_O + h^+^ → OH˙ + H^+^TAM–GEM + O_2_˙^−^ + H_2_ + OH + h^+^ → H_2_O + CO_2_ + C_3_H_10_NO_4_ + C_6_H_14_O + CH_3_COOH + C_6_H_10_O

Xie *et al.* synthesized Fe_3_O_4_/BiOI magnetic photocatalyst to break down sulfadiazine sodium, which is used to treat infectious disorders. As mentioned above, the photocatalyst activity was about 80%. It was discovered that adding iron oxide to the nanocomposite enhanced the separation of electrons and holes and gave it magnetic properties that made it easier to separate after drug degradation.^167^ Kumar *et al.* reported N–TiO_2_@SiO_2_@Fe_3_O_4_ magnetic nanocomposite for prescription photodegradation ibuprofen, carbamazepine, and benzophenone-3 (UV filter). The combination exhibited superior degradation against ibuprofen (94%), benzophenone-3 (93%), and carbamazepine (71%). The remarkable photodegradation efficiency has been ascribed to the modification of the electronic band structure caused by nitrogen doping. Following the degradation phenomena, the nanocomposite produced easy separation (95% in 20–25 minutes) under an external magnetic field.^168^ Khan *et al.* described the synthesis of a magnetic photocatalyst BiOBr/Fe_3_O_4_@SiO_2_ by solvothermal method for large-scale ibuprofen decontamination. Ibuprofen was nearly wholly (99%) degraded, and the nanocomposite was recycled using an electromagnet in under five minutes, demonstrating the photocatalyst's high stability.^169^ Additionally, Fe_3_O_4_/ZnO has been synthesized to eliminate four distinct antibiotics. All four antibiotics showed photocatalytic efficiencies of greater than 90%. Additionally, it was noted that the composite has comparable photocatalytic capability in subsequent reuse cycles, indicating its stability and reusability.^170^

Nasseh *et al.* reported a magnetic FeNi_3_@SiO_2_@ZnO nanocomposite to remove tamoxifen from wastewater successfully. ZnO was the primary component of this composite because of its appropriate bandgap. FeNi_3_ was a magnetic core, and the silica layer shielded the magnetic core from oxidation and light.^171^ There have been reports of the photocatalytic breakdown of anticancer medications using the magnetically separable nanocomposite Fe_3_O_4_/Au. Perhaps because of its magnetic iron core, the nanocomposite, as mentioned above, was easily separated by a simple magnet. However, gold nanoparticles stopped iron oxide from oxidizing and clumping together. After five cycles, the photocatalyst demonstrated extraordinary reusability and outstanding photocatalytic effectiveness for imipenem (84%) and imatinib (82%), both exposed to visible light.^172^ Ahmadpour *et al.* synthesized ZnFe_2_O_4_@TiO_2_/Cu using a solvothermal process and applied it against naproxen photodegradation. Comparing the ZnFe_2_O_4_@TiO_2_/Cu (80.73%) nanocomposite to ZnFe_2_O_4_@TiO_2_ (73.81%) and ZnFe_2_O_4_ (50.58%), the former demonstrated greater photoactivity. Cu particles have been associated with increasing ZnFe_2_O_4_@TiO_2_/Cu's photocatalytic effectiveness by decreasing electron/hole pair recombination and producing more free radicals.^173^

Wang *et al.* described the SnFe_2_O_4_/ZnFe_2_O_4_ photocatalyst that is active in visible light for the degradation of TC from aqueous media. Because of its ideal magnetic saturation value of 24.8 emu g^−1^, this magnetic heterojunction photocatalyst demonstrated 93.2% degrading ability and simple separation from aqueous solution. According to the theorized process, the holes in the valence band of SnFe_2_O_4_ were neutralized by the photoinduced electrons in ZnFe_2_O_4_, and these electron–hole pairs produced potent radicals that degraded tetracycline.^174^ Xu *et al.* revealed a Fe_3_O_4_/BiVO_4_/CdS nanocomposite that is visible light active and can photocatalytically break down tetracycline. They found that combining BiVO_4_ and CdS reduced the rate at which photoinduced charge carriers reacted, improving the nanocomposite's capacity to absorb light and increasing its surface area. On the other hand, Fe_3_O_4_ loading enabled its high separation *via* the magnetic field. These characteristics in one material have been linked with exceptional photocatalytic performance (87%) against tetracycline. Even after five runs, the stability and recyclability were determined to be above 81% in catalytic efficiency.^175^

Gabelica *et al.* showed that magnetite-based MNPs were effectively created utilizing a quick microwave-assisted synthesis method. The Fe_3_O_4_/SiO_2_/TiO_2_ nanocomposite was a potential option for ciprofloxacin degradation. After three consecutive replicates, Fe_3_O_4_/SiO_2_/TiO_2_ showed a remarkable 94% photodegradation efficiency and remained intact. The loading of Fe_3_O_4_ also confirmed the straightforward catalyst recovery using a magnet.^176^ ZnFe_2_O_4_/carbon derivatives (ZnFe_2_O_4_@CNTs, ZnFe_2_O_4_@GO, and ZnFe_2_O_4_@fullerene) have been reported for the photocatalytic degradation of norfloxacin. Carbon moieties enhanced light absorption in the visible spectrum because of the carbon derivatives' conjugated π–π structure and efficient electron trapping. However, ZnFe_2_O_4_@carbon nanotubes exhibited the highest degradation efficiency (91.36%) against norfloxacin because of the outstanding charge carrier separation efficiency brought about by the good interfacial connection between ZnFe_2_O_4_ and CNTs.^177^ Another visible light-active nanocomposite that had been synthesized for the efficient breakdown of 94% tetracycline is Fe_3_O_4_/g-C_3_N_4_/MoO_3_. The heterojunction creation between g-C_3_N_4_ and MoO_3_ has been attributed to this exceptional efficiency, as it has improved the segregation of charge carriers and increased the production of active species.^178^ Using a hydrothermal process, a different magnetic composite (g-C_3_N_4_/NiO/ZnO/Fe_3_O_4_) was synthesized for the photocatalytic degradation of esomeprazole at 95.05%. The synergistic contribution of the individual composite components that provided the structural and optical alterations is the only reason for this efficiency. The creation of light-induced charge carriers, their separation, and decreased recombination—all of which ultimately resulted in exceptional photocatalytic activity are appropriate for these modifications.^179^ According to the structural analysis, the synthesized AgCuFe_2_O_4_@MC/AC catalyst was created on a nanoscale with a crystalline structure, large surface area, appropriate magnetic characteristics, and optical activity. High efficiency in photodegrading TC, which is 90.91% from synthetic solutions and 87.17% from actual wastewater, was demonstrated by the synthesized catalyst under ideal circumstances (neutral pH, starting TC content of 5 mg L^−1^, nano-photocatalyst dosage of 0.5 g L^−1^, one and half hour). The results showed that the heterogeneous magnetic nano-photocatalyst AgCuFe_2_O_4_@MC/AC may be utilized successfully to clean wastewater from hospitals and pharmaceutical enterprises because of its strong capacity to degrade antibiotics, notably TC.^180^Table 2 represents the MNPs used in different environmental applications.

**Table 2:** MNPs for the removal of different contaminants

MNPs	Contaminant removed	Removal mechanism	Contact time	Removal efficiency	Saturation magnetization (*M*_s_)	Size (nm)	Reusability	Ref.
Humic acid functionalized Fe_3_O_4_	Carcinogenic malachite green dye	Adsorption	35 min	97%	70.05 emu g^−1^	5–15 nm	Up to five cycles for 85% removal	181
Combination of Fe_3_O_4_ and Fe_2_O_3_	Bromophenol blue (BPB)	Photocatalytic degradation	60 min	98%	—	10–80 nm	Up to three cycles for 95% removal	182
Superparamagnetic iron-oxide nanoparticles (SPION) and SPION/β-CD	Bisphenol A and malachite green dye	Photocatalytic degradation	10 min	82.5%	68.34 emu g^−1^	10–15 nm	70% removal for first five cycles	183
					39.21 emu g^−1^		Up to five cycles for 90.13% removal	
Epoxy-triazinetrione-functionalized MNPs (Fe_3_O_4_-ETT)	Malachite green (MG)	Adsorption	15 min	95%	30.7 emu g^−1^	25–52 nm	Up to six cycles for 61% removal	184
JC-Fe_3_O_4_ and CT-Fe_3_O_4_ NPs	Organic dyes	Adsorption	120 min	99%	38.46 emu g^−1^	20–42 nm	—	185
					34.35 emu g^−1^	26–35 nm		
Fe_3_O_4_@NiO core shell MNPs	Alizarin red S dye	Adsorption	60 min	96.58%	24.7 emu g^−1^	65 ± 10 nm	Up to six cycles for 43.93% removal	186
MAPE-mediated iron oxide nanoparticles (MION)	Sunset yellow dye	Adsorption	180 min	92%	44.14 emu g^−1^	20–60 nm	Up to five cycles for 75.4% recovery	187
Yeast cells immobilized Fe_3_O_4_ MNPs	Methyl orange dye	Biosorption	140 min	96.52%	—	87.46–231.4 nm	—	188
Copper-doped ZrO_2_ MNPs	Methyl orange dye	Photocatalytic degradation	100 min	98%	—	20–45 nm	Up to four cycles for 90% removal	189
MCPEI (polyethyleneimine and magnetic nanoparticle)	Black 5 dye	Adsorption	180 min	100%	3.3 emu g^−1^ (before adsorption)	1.92 nm (pore size)	Up to five cycles for 48% removal	190
					3.2 emu g^−1^ (after adsorption)			
Zeolite/Fe_3_O_4_	Basic violet 16 (BV16)	Adsorption	45 min	99%	18.11 emu g^−1^	1–4 nm	Up to five cycles for 64.17% removal	191
Fe_3_O_4_ MNPs	Methylene blue (MB)	Adsorption	60 min	88.8%	49.48 emu g^−1^	21–32 nm	Up to four cycles for 77% removal	192
Magnesium oxide modified magnetic nanoparticles (MgO@MNPs)	Eriochrome black T (EBT) dye	Adsorption	5 min	98%	11.70 emu g^−1^ (before adsorption)	33.33–50.80 nm	Up to seven cycles for 96% removal	193
					10.62 emu g^−1^ (after adsorption)			
Fe_3_O_4_ MNPs	Anionic azo dye	Adsorption	120 min	99.99%	—	50 nm	—	194
Magnetic core–shell Fe_3_O_4_@SiO_2_ nanoparticles	Methyl orange	Adsorption	30 min	83%	72.08 emu g^−1^	120 nm	Reusable up to 5 cycles	195
Magnesium doped CoFe_2_O_4_ NPs	Methylene blue (MB) dye	Adsorption	30 min	98.6%	15.2971 emu g^−1^	7.9 nm	Up to five cycles 82.5% removal for MB and 79.6% removal for RhB	196
	Rhodamine B (RhB) dye			95.3%				
Nanoparticles of titanomagnetite (NTM)	Methylene blue (MB)	Adsorption	20 min	—	62 emu g^−1^	35 nm	Reusable for up to three successive cycles	197
Fe_3_O_4_@Ag@MESNa	Hg(ii)	Adsorption	30 s	100%	—	—	Up to two cycles for 100% removal	198
Magnetic eggshell membrane (MESM)	Lead	Adsorption	48 h	95%	66 emu g^−1^	∼50 nm	Reusable	199
Fe_3_O_4_@EPS	PO_4_^2−^	Adsorption	13 h	91%	5.0 emu g^−1^	10–20 nm	—	200
Magnetic tubular carbon nanofibers (MTCFs)	Cu(ii)	Adsorption	10 min	99.9 ± 0.1%	10.65 emu g^−1^	25–110 nm	Up to six cycles for 85% removal	201
Magnetic nanocomposite activated hydrochar (Fe_3_O_4_-ACH)	Cr(vi)	Adsorption	120 min	94.1%	0.110334 emu g^−1^	0.7–1.5 mm	Up to five cycles for 82.7% removal	202
Cabuya fibers impregnated magnetite nanoparticles (FC–MNPs) and MNPs	Hg(ii)	Adsorption	4 h	93%	—	19 nm	—	203
JC-Fe_3_O_4_ and CT-Fe_3_O_4_ NPs	Co^2+^ and Cu^2+^	Adsorption	120 min	90%	38.46 emu g^−1^	20–42 nm	—	185
					34.35 emu g^−1^	26–35 nm		
Hyperbranched polyglycerol HPG–MNPs	Ni, Cu and Al	Adsorption	130 min	94%	−77 to +77 emu g^−1^	21–30 nm	Up to nine cycles for 90% removal	204
Chitosan-coated magnetic nanoparticles (cMNPs)	Bio-refinery wastewater containing heavy metals (Cu, Cr, and As)	Adsorption	90 min	Cu (42.2%), Cr (18.7%), and As (2.44%)	26.96 emu g^−1^	—	Up to five cycles for 20% of initial removal efficiency	205
Superparamagnetic Fe_3_O_4_@SiO_2_@GLYMO(S)-en	Pb^2+^ and Cd^2+^	Adsorption	55 min	—	40 emu g^−1^	16 nm	Up to five cycles for 90% removal	206
Epoxy-triazinetrione-functionalized MNPs (Fe_3_O_4_-ETT)	Pb(ii)	Adsorption	15 min	95%	30.7 emu g^−1^	25–52 nm	Up to six cycles for 61% removal	184
Eucalyptus leaf extracts (EL–MNP@zeolite)	PO_4_^2−^	Adsorption	30 min	99.8%	—	60 nm	—	207
	NH_4_^+^			43.3%				
MNP@SiO_2_–PEI–DTPA	Pb^2+^	Adsorption	72 h	>90%	85 emu g^−1^	25–150 nm	Up to five cycles for 80% removal	208
	Cd^2+^							
Nano-chitosan coated MNPs	Pb(ii)	Adsorption	10–30 min	57.6–95.5%	—	9.32–20.57 nm	Up to three cycles for 96% removal	209
	Cu(ii)							
	Cd(ii)							
C–CoFe_3_O_4_/N, S-BC	Pb^2+^	Adsorption	30 min	99.1%	—	—	Up to eight cycles with >80% removal efficiency	210
Fe_3_O_4_/CNTs nanocomposites	Pb^2+^	Adsorption	60 min	91.64%	8.9 and 16.4 A m^2^ kg^−1^	2–30 nm	Reusable up to six cycles	211
Ionic-liquid modified MNPs (Fe_3_O_4_@SiO_2_–[C_3_C_1_Im]Cl NPs)	Platinum (Pt)	Adsorption	15 min	22–24%	51 emu g^−1^	65 nm	—	212
	Palladium (Pd)		1 h	90–93%				
			6 h					
DES/GO–Fe_3_O_4_ nanohybrid	Pb(ii)	Adsorption	20 min	80%	—	6–30 nm	—	213
Magnetic nanoparticles coated mixed fungal biomass (MNP–FB)	Cr(vi)	Adsorption	60 min	99.4%	—	—	—	214
AG-g-PAN/Cu Fe_2_O_4_	Pd(ii)	Adsorption	30–120 min	97.01%	12 emu g^−1^	37 nm	Up to three cycles for 93.98% removal	215
Fe_3_O_4_ and Fe_3_O_4_–chitosan NPs	Pb(ii)	Adsorption	31.2 min	69.02 and 89.54%	51.22 and 27.86 emu g^−1^	30 nm	Up to five cycles for 59.43% removal	216
Fe_3_O_4_@DA–DMSA magnetic nanoparticles (FDDMs)	Pb^2+^	Adsorption	45 min	90.11%	21.50 emu g^−1^	446 nm	—	217
	Cu^2+^			40.61%				
	Cd^2+^			13.87%				
Core shell magnetic nanoparticles (cMNPs)	As(v)	Adsorption	10 min	97.5%	36.4 emu g^−1^	4.9 nm	Up to five cycles for 80% removal	218
Fe_3_O_4_@ZIF-8 and Fe_3_O_4_@SiO_2_@ZIF-8	Pb^2+^	Adsorption	15 min	93.61%	—	110 ± 10 nm and 80 ± 10 nm	—	219
				99.2%				
Magnetic hybrid alumina nanoparticles (MHAI-NPsP)	Cu(ii)	Adsorption	60 min	85%	—	—	—	220
Gum arabic magnetic nanoparticles (GA–MNPs)	Ciprofloxacin (CIP)	Adsorption	120 min	96.30%	—	—	Up to seven cycles for initial removal efficiency of 96.34%	221
Fe_3_O_4_-ACLM	Ciprofloxacin (CIP)	Adsorption	75 min	100%	37.6 emu g^−1^	9.8 ± 1.5 nm	Up to six cycles for 90% removal	222
C–CoFe_3_O_4_/N, S-BC	Ciprofloxacin (CIP)	Adsorption	30 min	94.7%	—	—	Up to eight cycles with >80% removal efficiency	210
Fe_3_O_4_@MIL-125-NH_2_	(Pesticides) carbaryl	Photocatalytic degradation	150 min	91%	—	24.57 nm	—	223
	Methomyl			98%				
Activated carbon magnetic nanoparticles (AC–MNPs)	Metronidazole (MTZ)	Adsorption	90 min	100%	24.93 emu g^−1^	20–35 nm	Up to five cycles for 90.5% removal	224
PAC@Fe_3_O_4_–MN	Ciprofloxacin (CIP)	Adsorption	60 min	100%	51.5 emu g^−1^	<80 nm	Up to eight cycles for 96% removal	225
MOM-Fe_3_O_4_	Pharmaceutical substance (metformin)	Adsorption	720 min	93.9%	—	—	—	226
Fe_3_O_4_/clinoptilolite	Tetracycline	Adsorption	20 s	98.6%	30.0 emu g^−1^	60–80 nm	Up to four cycles for 80.6% removal	227
Fe_3_O_4_@gly@indole@CuNO_3_ magnetic nanoparticle (FGICu)	Tetracycline	Adsorption	60 min	95%	38.4 emu g^−1^	17.8 nm	Up to five cycles for 70% removal	228
Magnetic sporopollenin supported magnesium nanoparticles (MSP@MgO)	Tetracycline	Adsorption	180 min	98.05%	∼32 emu	—	Up to 15 cycles for 84.72% removal	229
Magnetic Ho_2_MoO_6_/Fe_2_O_3_	Tetracycline	Adsorption	90 min	99.96%	14.82 emu g^−1^	25–50 nm	Up to five consecutive cycles for 79.1% removal	230
FeNi_3_@SiO_2_@TiO_2_	Humic acid (HA)	Photocatalytic degradation	30 min	100%	18.84 emu g^−1^	20–30 nm	—	231
Nano porous Co_2_O_3_/Cu_2_O_3_:Al_2_O_3_:SiO_2_	*E. faecalis*	Disinfection	05 min	100%	0.157–0.24 emu g^−1^	23–31 nm	—	232
Chitosan-coated magnetic nanoparticles (cMNPs)	Lignocellulosic biorefinery containing phenol	Adsorption	90 min	46.2%	26.96 emu g^−1^	—	Up to five cycles for 20% of initial removal efficiency	205
Novel MNP–alum conjugate	Natural organic matter	Adsorption cum enhanced coagulation–flocculation	30 min	98.7%	—	20–30 nm	Up to five cycles with ∼91% removal	233
NFO@SiO_2_@APTS	Acetaminophen	Adsorption	30 min	94%	22 emu g^−1^	130–150 nm	Up to four cycles for 89% removal	234
Magnetic mesoporous silica microspheres (MSMS)	Acetaminophen	Adsorption	30 min	97.4%	8.3 emu g^−1^	26 nm	Reusable for up to 14 cycles	235
Fe_3_O_4_/Douglas fir biochar	Acetylsalicylic acid	Adsorption	5 min	70%	—	12.3 ± 7.1 nm	Reusable for up to five cycles	236
NFO@SiO_2_@APTS	Ibuprofen	Adsorption	30 min	97%	22 emu g^−1^	130–150 nm	Up to three cycles for 92% removal	234
Magnetic activated carbon–Fe_3_O_4_ (AC–Fe_3_O_4_)	Promazine	Adsorption	06 min	99.97%	—	20 nm	Up to five cycles for 99% removal	237
Magnetic silica-based nanoadsorbents (MMST and MMST-Ph)	Pharmaceutical substance	Adsorption	200 min	80%	16.0 emu g^−1^ (MMST)	3.75 nm (pore size)	Up to eight cycles for 100% removal	238
					18.6 emu g^−1^ (MMST-Ph)			
MNPs coated with rhamnolipids (Rh-cMNP)	Acetaminophen	Adsorption	60 min	96.7%	16.5 emu g^−1^ (before adsorption)	—	Up to eight cycles for	239
					8.2 emu g^−1^ (after adsorption)			
Fe_3_O_4_@GNP	Ketoprofen (KP)	Adsorption	30 min	For wastewater (65.0–83.6%)	—	50 nm	Reusable for up to five cycles	240
	Ibuprofen (IP)			For tap water (67.1–90.0%)				
Rape straw biomass fiber-β-CD–Fe_3_O_4_ (RSBCDF)	Ibuprofen (IP)	Adsorption	5–150 min	94.42%	—	150–250 nm	Up to five cycles for 73.56%	241
Fe_3_O_4_@GNP	Naproxen (NX)	Adsorption	30 min	For wastewater (65.0–83.6%)	—	50 nm	Reusable for up to five cycles	240
	Diclofenac sodium salt (DF)			For tap water (67.1–90.0%)				
Biochar/MgFe_2_O_4_	Levofloxacin (LFX)	Adsorption	30 min	Up to 80%	66.815 emu g^−1^	—	Up to four cycles for 74.25%	242
Fe_3_O_4_/Douglas fir biochar	Caffeine	Adsorption	5 min	70%	—	12.3 ± 7.1 nm	Reusable for up to five cycles	236
3D-GC/MGO–SO_3_H	Tetracycline	Adsorption	1 h	85%	55.4 emu g^−1^	80–90 nm	Up to five cycles for 85% removal	243
Magnetic polydopamine-chitosan modified adsorbent (MPDA-CS)	Diclofenac sodium (DCFS)	Adsorption	40 min	96.38%	—	290.2 nm	Up to three cycles for 94% removal	244
Fe_3_O_4_-gINPs	Levofloxacin (LEV)	Adsorption	24 h	86.15%	—	14.34 nm	Up to four cycles for 80.47% removal	245
Magnetic molecularly imprinted polymers (MMIP)	Norfloxacin	Adsorption	—	—	—	—	Reusable	246
Fe–TiO_2_@Fe_3_O_4_	2,4-Dichlorophenol	Sono-catalytic degradation	90 min	94%	25 emu g^−1^	15.05–18.12 nm	Up to five cycles for 91% removal	247
Fe_3_O_4_@Phe NPs	Ciprofloxacin (CIP)	Adsorption	30 min	62.5%	60 emu g^−1^	150–270 nm	Up to four cycles for 80% removal	248
Zn_0.5_Ni_0.5_FeCrO_4_ MNPs	4-Nitrophenol	Photo-degradation	180 min and 150 min	95% and 80%	7.06 emu g^−1^	25–35 nm	Up to four cycles reusable	249
	Aniline							
Polyvinylidene fluoride (PVDF)/Fe_3_O_4_@carboxymethyl cellulose	Oil sludge (oleic acid)	Sorption	30 min	—	62–55 emu g^−1^	20 nm	Up to five cycles reusable	250
Magnetic Janus nanoparticles (M-Janus NPs)	Cooking oil and crude oil	Phase-separation	15 min	96%	—	2 mm	Up to five cycles for 90% removal	251
Surface-modified MNPs, AU–MNPs, and AP–MNPs	Oil spills (crude oil)	Adsorption	3–20 min	98%	40.23 emu g^−1^	141.8 nm	Up to four cycles for 89% removal	252
					43.85 emu g^−1^	133.4 nm		
VCL/Fe_3_O_4_ and VAA/Fe_3_O_4_	Crude oil	Adsorption	5–20 min	98%	54.6 emu g^−1^	117.5 nm	Up to four cycles for 80% removal	253
				99.3%	49.4 emu g^−1^	173.6 nm		
Fe_3_O_4_ MNP	Crude oil	Adsorption	5–30 min	94%	—	3 nm	Up to nine cycles for 64% removal	254
				83%		15–20 nm		
Fe_3_O_4_ MNP	Crude oil	Adsorption	—	98.6%	51 ± 0.4 emu g^−1^	5–10 nm	Up to 7 cycles for 90% removal	255
Nanoparticles of titanomagnetite (NTM)	Crude oil spills	Adsorption	20 min	—	62 emu g^−1^	35 nm	Reusable for up to three successive cycles	197
HAN–Fe_3_O_4_	Crude oil	Adsorption	8 min	100%	—	10 nm	Up to five cycles for reusable	256
HAT–Fe_3_O_4_				89%		30 nm		
Magnetic corncob powder (CoFe_2_O_4_)	Crude oil (API = 23.3°)	Adsorption	30 min	—	32 emu g^−1^	5–50 nm	Up to 10th cycle with 41.98 g per g oil sorption capacity	257
	Crude oil (API = 29.7°)							
	Fresh motor oil							
	Used motor oil							
	Kerosene							
DOTAC-coated MNPs (M3)	Oil spills	Adsorption	3 min	94%	∼26.0 emu g^−1^	—	Up to 7 cycles in W/O emulsion with >90% efficiency	258
MNP–S@CTAB	Oil	Adsorption	20 min	100%	—	15–20 nm	Up to 10th cycles with 19 mg g^−1^ adsorption efficiency	259
						50–100 nm		
Silica coated ferroferric oxide (Fe_3_O_4_@SiO_2_)	Emulsified oil	Transformation	05 min	98%	69.1 emu g^−1^	200 nm	Up to five cycles for 95% removal	260

### 3.5 Scalability challenges

Scalability challenges encompass several factors, including the recovery rates and reusability of nanoparticles, treatment costs, and the economic feasibility of the processes involved, as well as the influence of complex environmental conditions such as high salinity and low temperatures. Among these, the reusability of MNPs stands out as a critical aspect. The economic viability of MNPs is significantly tied to their ability to be reused; however, issues like surface fouling and structural degradation can diminish their adsorption efficiency after multiple cycles.^261^ For MNPs to be commercially feasible in large-scale applications, it is essential to develop more sustainable and efficient regeneration methods. Enhancing the reusability of MNPs can be achieved by exploring strategies that minimize nanoparticle degradation, such as enzymatic treatments or low-temperature regeneration techniques.^18^ Metal–organic frameworks and metal-based adsorbents exhibit high adsorption capacities due to the nature of adsorption through metal complexation. However, challenges remain regarding the recovery, stability, recycling, and potential metal leaching associated with these materials.^262^ Furthermore, the economic feasibility of large-scale applications may be hindered by the energy costs associated with magnetic recovery, necessitating further optimization.^263^ Recent literature reveals a shift from traditional carbon-based materials to various promising alternatives that are not only more cost-effective for industrial applications but also demonstrate comparable performance to carbon-based adsorbents.^264^

Despite the exploration of advanced adsorbent materials such as ceramics, MOFs, graphene oxide, and composite adsorbents in small-scale studies using simulated hospital wastewater or simplified water sources, their long-term effectiveness in real hospital wastewater (HWW) remains largely unexamined. Currently, only granular and powdered activated carbons have seen widespread application. Challenges such as high operational costs, non-specific adsorption, degradation of material and adsorption capacity due to frequent regeneration, and the management of spent adsorbents pose significant economic and environmental hurdles. To address these issues, the development of innovative adsorbent materials that offer high adsorption capacity, durability, and ease of regeneration is essential for effective full-scale wastewater treatment.^265^ While MOF-derived magnetic nanocomposites offer numerous advantages, such as significant structural and chemical flexibility, the costs involved in their synthesis still require optimization. This includes expenses related to raw materials, like metallic salts and organic binders, as well as processing methods that often utilize non-recyclable organic solvents and activation techniques. One potential solution is to adopt aqueous synthesis in place of solvothermal methods, which could lead to a cost reduction of approximately US$ 35 for every US$ 13 spent on synthesizing MOFs at a large scale.^266^ For industrial applications to be economically viable, the cost of these materials should ideally be below $10 per kilogram. Consequently, it is essential to focus on less expensive organic ligands, advocating for the use of shorter and smaller binders rather than more expensive longer or aromatic alternatives.^267^ Nevertheless, there is a notable gap in the literature regarding cost evaluations, which are essential for assessing economic viability at scale. Research is increasingly focused on analyzing the economic feasibility of pollution control methods that incorporate these nanocomposites, taking into account production, operational costs, waste management, and material regeneration.^268^

Despite the advantages offered by nanostructured adsorbents, several challenges hinder their broader application, primarily their high production costs and difficulties in scaling.^269^ The expense associated with manufacturing these materials renders them impractical for large-scale water treatment initiatives. Additionally, while nanostructured adsorbents have demonstrated effectiveness in laboratory settings, translating this success to larger systems remains problematic. Key considerations, such as the efficient distribution and maintenance of these adsorbents in real-world applications, must be thoroughly addressed before they can be adopted on a wider scale.^270^

In light of these challenges, further research and development are essential to address the existing issues and enhance the potential benefits of nanostructured adsorbents in water treatment applications. These materials have proven effective in various wastewater treatment scenarios, including the removal of organic pollutants, dyes, and heavy metals. For instance, surface-modified iron oxide nanoparticles have successfully eliminated toxic elements such as lead and arsenic from industrial wastewater. Additionally, carbon-based nanostructured materials, including carbon nanotubes and modified graphene, have demonstrated significant efficacy in extracting organic contaminants and dyes due to their high surface area and adsorption capacity. These practical applications underscore the versatility and effectiveness of nanostructured adsorbents in tackling diverse challenges in wastewater treatment.

A thorough research into the use of nanostructured adsorbents for the continuous treatment of sewage, industrial effluents, and polluted water bodies is essential. While surface-functionalized nanostructured adsorbents have demonstrated effectiveness in laboratory settings, their application in industrial environments is limited due to high costs and complex modification processes. To promote advancements in wastewater treatment technologies, it is crucial to focus on developing cost-effective preparation methods, selectively modifying surface functional groups, and further investigating pollutant removal mechanisms, recovery of valuable elements from wastewater, and the recycling of adsorbents.^271^ Scalability challenges such as high salinity in wastewater and low temperatures can significantly hinder the effectiveness of MNPs in large-scale environmental remediation efforts. These conditions can diminish treatment efficiency, compromise material stability, and reduce adsorption capabilities. The presence of elevated salt concentrations, commonly found in oilfield brines, desalination processes, and various industrial effluents, poses a particular obstacle to the scalability of magnetic nanohybrid materials. Salts like NaCl, MgCl_2_, and CaSO_4_ interfere with the electrostatic interactions between contaminants and nanomaterials, disrupting surface chemistry and leading to decreased adsorption effectiveness due to competition for active sites.^272^ Furthermore, chloride ions can promote the leaching of iron ions from magnetite, thereby destabilizing metal oxide nanoparticles and affecting their durability and magnetic separability. Additionally, elevated ionic strength can lead to the aggregation of nanoparticles, further reducing their reactivity and active surface area.^273^

The effectiveness of MNPs in environmental remediation can be significantly influenced by low ambient temperatures. Key processes such as diffusion rates, redox reactions, and the kinetics of pollutant adsorption, which are vital for nanohybrid-based remediation, are all temperature-dependent. For instance, semiconductor–metal nanohybrid-mediated photocatalytic degradation is notably less efficient at lower temperatures due to reduced activation energy and slower electron transfer rates. Additionally, colder temperatures increase the viscosity of wastewater, hindering the mobility of nanoparticles and their capacity to engage with contaminants. These thermal constraints pose challenges to the sustainability and cost-effectiveness of large-scale applications, making the deployment of MNPs in temperate or cold regions less feasible without additional energy support.^274^

The large-scale application of MNPs in real-world settings faces significant challenges due to the interplay of low temperatures and high salinity. These conditions can slow down reaction kinetics and promote the aggregation of nanoparticles, thereby reducing their effectiveness in pollutant removal. To address these issues, it is essential to develop more robust nanohybrids that maintain their dispersibility and catalytic performance across diverse environmental conditions, such as surface-functionalized or polymer-stabilized MNPs. Furthermore, this situation underscores the importance of conducting pilot studies and implementing site-specific adaptive designs before embarking on large-scale remediation efforts.^275^ Scalability is essential for enhancing productivity and transitioning laboratory-scale synthesis to large-scale operations, particularly in both batch and continuous processes. A key engineering challenge in designing modular reactors for large-scale water treatment lies in achieving uniform flow dynamics and treatment efficacy across all modules. Typically arranged in parallel or series within decentralized systems, these modular reactors face theoretical difficulties in ensuring consistent flow distribution and hydraulic retention time. Such variations can lead to overloaded or underutilized reactor zones, ultimately diminishing pollutant removal efficiency. Additionally, non-uniform utilization of reactive media, such as MNPs, can hinder effective magnetic separation and result in unequal pollutant exposure, especially in high-flow industrial or municipal environments.^276^ While the application of nanoparticles in real-world scenarios is increasingly viable, challenges remain regarding their production and broader implementation. Most research is still conducted at the bench level, necessitating pilot-scale studies to validate findings. The scaling up of green nanoparticle production is promising, as larger-scale operations can provide more accurate data on cost-effectiveness, repeatability, and efficiency.

### 3.6 Environmental impact assessment

The increasing interest in magnetic nanohybrid materials highlights their unique characteristics and wide-ranging applications, especially in environmental remediation. While extensive research has been conducted on the efficacy and uses of MNPs, it is crucial to also assess their potential environmental impacts. An effective environmental impact assessment for MNPs should concentrate on three primary aspects: the release of harmful substances during their application, their influence on ecosystems, and the strategies for ensuring safe ecological remediation. A unified scientific framework is essential for researchers to evaluate sustainable practices associated with MNP applications. It is vital to investigate the potentially harmful substances that may be released during MNP usage, as their production involves various chemical compounds, some of which could be toxic. Furthermore, the degradation of MNPs can result in the release of nanoparticles, metal ions, or other byproducts that may endanger human health and the environment. For instance, a study by Osman *et al.* focused on synthesizing a highly active magnetic sorbent composite from mixed plastic and biomass waste, employing Life Cycle Assessment (LCA) to evaluate the environmental impacts of producing the magnetic char composite adsorbent, adhering to the methodologies and standards set forth in ISO 14040:2006 and ISO 14044:2006.^277,278^

Ferric iron is primarily transported in the body by transferrin, which interacts with transferrin receptors on cell surfaces, while its storage occurs mainly in ferritin proteins within cells. Initially, MNPs were considered non-toxic due to their ability to release ferric iron into normal iron metabolism. However, their diminutive size enables them to accumulate in high concentrations within cells, complicating their elimination from the body.^279^ This accumulation raises significant concerns, particularly because excess free iron can produce harmful free radicals that threaten sensitive tissues, such as the brain. Research by Bucak *et al.* has shown that toxicity can emerge when serum proteins adhere to the surfaces of MNPs, thereby modifying the cellular environment. To mitigate this risk, coating MNPs with biocompatible materials can effectively reduce protein binding and surface interactions, ultimately lowering their toxicity.^280^

A comprehensive examination of the effects of MNPs on ecosystems is crucial due to their distinctive magnetic properties and large surface area, which allow them to interact with various environmental components such as water, soil, air, and living organisms. Although MNPs can effectively break down water pollutants, their degradation products may pose risks to aquatic ecosystems and disrupt food webs. Furthermore, the application of MNPs in soil remediation can modify microbial communities, potentially hindering plant growth. To assess these impacts, thorough ecotoxicological evaluations are essential. Establishing best practices and guidelines for the safe use of MNPs in environmental remediation is vital, emphasizing the principles of green chemistry and sustainable engineering in their synthesis and design. This approach should focus on utilizing sustainable chemical materials that foster non-toxic interactions and incorporate recyclable, eco-friendly processes to reduce harmful byproducts. Techniques such as coating and functionalization can enhance the stability and biocompatibility of MNPs, thereby lowering their toxicity. The integration of biocompatible polymers and organic ligands can improve the distribution of MNPs in environmental media and prevent aggregation, which in turn minimizes their chemical reactivity and potential negative effects.

The effective and safe integration of MNPs necessitates collaboration among researchers, industry stakeholders, and regulatory bodies to ensure sustainable practices. It is crucial to disseminate research findings on the environmental impacts of MNPs through scientific journals, conferences, and user-friendly online platforms. Industry stakeholders are encouraged to adopt best practices for the synthesis, management, and disposal of MNPs, adhering to established standards and guidelines. Regulatory agencies must create clear regulations that outline the proper control of MNPs during environmental impact assessments at the company level. Additionally, public education and community engagement are vital for raising awareness about both the beneficial and harmful aspects of MNPs, empowering individuals to make informed decisions.

**Fig. 12:**
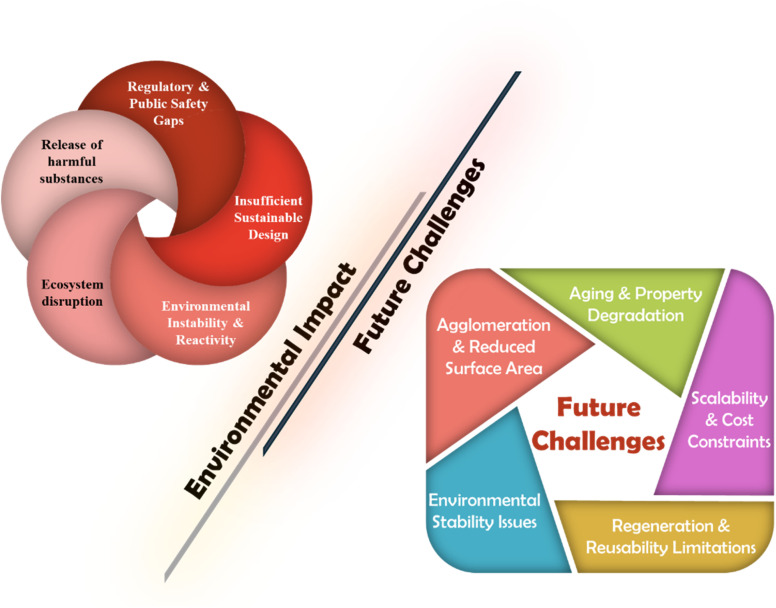
Pictorial illustration of environmental impact and future challenges.

Environmental assessments of magnetic nanohybrid materials are essential for their sustainable application in environmental remediation. By carefully evaluating the potential release of toxic substances and their ecological effects, we can reduce the environmental risks linked to magnetic nanoparticles through the implementation of appropriate safety measures. This thorough evaluation framework not only bolsters the safety and security of these materials but also establishes a scientific foundation for their responsible and sustainable utilization. Continued collaboration in research and regulatory initiatives is vital to enhance our understanding of the environmental consequences of magnetic nanoparticles, facilitating the creation of safe and sustainable practices for their ecological use. Fig. 12 illustrates the environmental impact and future challenges associated with these materials.

## 4. Conclusions and future directions

Magnetic nanohybrid materials (MNMs) have gained recognition as effective agents for environmental remediation due to their distinctive properties, including high surface reactivity, magnetic separability, and customizable functionalities for pollutant extraction. Their versatility allows for applications in water purification, soil decontamination, and air filtration, as they can efficiently adsorb, catalyze, or degrade various contaminants. Nevertheless, challenges such as agglomeration, long-term stability in environmental settings, scalability of production, and cost-effectiveness pose significant barriers to their broader implementation. To overcome these issues, future research should focus on innovative material designs and the integration of advanced technologies. A particularly promising avenue is the creation of stimuli-responsive MNMs, which can adjust to changes in environmental conditions, thereby enhancing pollutant capture and regeneration. Furthermore, the application of artificial intelligence and machine learning holds the potential to transform the optimization of nanomaterials by accurately predicting synthesis parameters, surface modifications, and interactions with pollutants, ultimately facilitating closed-loop systems for real-time performance improvements.

A vital area of focus is the enhancement of MNMs' durability and reusability through advanced stabilization methods, such as atomic-layer deposition and bio-inspired coatings like polydopamine and cellulose derivatives, which help reduce aging and aggregation. Additionally, employing core–shell structures with protective layers can further bolster their chemical and magnetic stability in challenging environments. For broader application, it is essential to investigate green synthesis techniques that utilize plant extracts or microbial processes, thereby minimizing the use of hazardous chemicals and decreasing production costs. Moreover, the integration of MNMs into hybrid photocatalytic systems presents an opportunity for solar-driven degradation of persistent pollutants, facilitating easy magnetic recovery and effectively linking photocatalysis with practical remediation efforts.

In conclusion, while MNMs hold immense promise for addressing environmental pollution, their transition from laboratory innovations to practical applications, a multidisciplinary approach is essential. Emphasizing smart material design, AI-driven optimization, sustainable manufacturing, and hybrid catalytic systems will enable researchers to address current challenges and fully harness the capabilities of these nanomaterials. Collaborative efforts among chemists, engineers, and data scientists are crucial for refining synthesis methods, enhancing stability, and ensuring economic feasibility. With focused advancements in these domains, MNMs have the potential to significantly contribute to sustainable environmental remediation, fostering cleaner ecosystems and supporting a circular economy.

## Conflicts of interest

The authors declare no conflict of interest regarding the publication of this manuscript.

## Data Availability

Data are available within the manuscript in the form of figures and tables. No primary research results, software or code have been included and no new data were generated or analyzed as part of this review.
